# Genomic and transcriptomic analysis of breast cancer identifies novel signatures associated with response to neoadjuvant chemotherapy

**DOI:** 10.1186/s13073-024-01286-8

**Published:** 2024-01-12

**Authors:** Gengshen Yin, Liyuan Liu, Ting Yu, Lixiang Yu, Man Feng, Chengjun Zhou, Xiaoying Wang, Guoxin Teng, Zhongbing Ma, Wenzhong Zhou, Chunmiao Ye, Jialin Zhang, Changhua Ji, Linfeng Zhao, Peng Zhou, Yaxun Guo, Xingchen Meng, Qinye Fu, Qiang Zhang, Liang Li, Fei Zhou, Chao Zheng, Yujuan Xiang, Mingming Guo, Yongjiu Wang, Fei Wang, Shuya Huang, Zhigang Yu

**Affiliations:** 1 Department of Breast Surgery, The Second Hospital of Shandong University, Jinan, 250033 China; 2 Institute of Translational Medicine of Breast Disease Prevention and Treatment, Shandong University, Jinan, 250033 China; 3 Shandong Provincial Engineering Laboratory of Translational Research On Prevention and Treatment of Breast Disease, Jinan, 250033 China; 4 Research Center for Mathematics and Interdisciplinary Sciences, Shandong University, Qingdao, 266237 China; 5 Department of Pathology, The Third Affiliated Hospital of Shandong First Medical University (Affiliated Hospital of Shandong Academy of Medical Sciences), Jinan, 250031 China; 6 Department of Pathology, The Second Hospital of Shandong University, Jinan, 250033 China; 7 Institute of Medical Sciences, The Second Hospital of Shandong University, Jinan, 250033 China; 8 Department of Breast Surgery, Weifang People’s Hospital, Weifang, 261041 China

**Keywords:** Breast cancer, Neoadjuvant chemotherapy, Genomic, Transcriptomic, Pathological response, Prognosis

## Abstract

**Background:**

Neoadjuvant chemotherapy (NAC) has become a standard treatment strategy for breast cancer (BC). However, owing to the high heterogeneity of these tumors, it is unclear which patient population most likely benefit from NAC. Multi-omics offer an improved approach to uncovering genomic and transcriptomic changes before and after NAC in BC and to identifying molecular features associated with NAC sensitivity.

**Methods:**

We performed whole-exome and RNA sequencing on 233 samples (including matched pre- and post-treatment tumors) from 50 BC patients with rigorously defined responses to NAC and analyzed changes in the multi-omics landscape. Molecular features associated with NAC response were identified and validated in a larger internal, and two external validation cohorts, as well as in vitro experiments.

**Results:**

The most frequently altered genes were *TP53*, *TTN*, and *MUC16* in both pre- and post-treatment tumors. In comparison with pre-treatment tumors, there was a significant decrease in C > A transversion mutations in post-treatment tumors (*P* = 0.020). NAC significantly decreased the mutation rate (*P* = 0.006) of the DNA repair pathway and gene expression levels (FDR = 0.007) in this pathway. NAC also significantly changed the expression level of immune checkpoint genes and the abundance of tumor-infiltrating immune and stroma cells, including B cells, activated dendritic cells, γδT cells, M2 macrophages and endothelial cells. Furthermore, there was a higher rate of C > T substitutions in NAC nonresponsive tumors than responsive ones, especially when the substitution site was flanked by C and G. Importantly, there was a unique amplified region at 8p11.23 (containing *ADGRA2* and *ADRB3*) and a deleted region at 3p13 (harboring *FOXP1*) in NAC nonresponsive and responsive tumors, respectively. Particularly, the *CDKAL1* missense variant P409L (p.Pro409Leu, c.1226C > T) decreased BC cell sensitivity to docetaxel, and *ADGRA2* or *ADRB3* gene amplifications were associated with worse NAC response and poor prognosis in BC patients.

**Conclusions:**

Our study has revealed genomic and transcriptomic landscape changes following NAC in BC, and identified novel biomarkers (*CDKAL1*_*P409L*_, *ADGRA2* and *ADRB3*) underlying chemotherapy resistance and poor prognosis, which could guide the development of personalized treatments for BC.

**Supplementary Information:**

The online version contains supplementary material available at 10.1186/s13073-024-01286-8.

## Background

Breast cancer (BC) is the most frequent malignancy and the leading cause of cancer-related deaths among women worldwide [1]. Neoadjuvant chemotherapy (NAC) is currently the standard treatment for high-risk early-stage, locally advanced or inoperable BC. NAC is performed before surgery to reduce tumor burden and test the sensitivity of BC to treatment. Previous studies have indicated that response to NAC is significantly associated with the prognosis of BC patients [2, 3]. However, the benefit varies from patient to patient.

If tumors are sensitive to NAC, optimal treatment strategies can be used to improve the outcome. It has been demonstrated that patients with pathologic complete response (pCR) to NAC improve disease-free survival (DFS) and overall survival (OS) [2, 4–6]. This has made achieving pCR one of the main objectives of NAC. Unfortunately, pCR occurs only in a small proportion of BC patients, and differs significantly according to tumor subtypes [7]. Therefore, it is critical to identify patients who are most likely to benefit from NAC. To date, several clinical biomarkers have been exploited in clinics to assess NAC response, including Ki-67 expression, tumor size and molecular subtype. Multiple predictive molecular biomarkers have also been investigated in clinical trials involving neoadjuvant therapies. It has been shown that *BRCA1/2* mutation status leads to a better response to NAC in BC whereas *PIK3CA* and *TEKT4* mutations are associated with resistance to neoadjuvant therapy, including chemotherapy and targeted therapy [8–12]. Previous studies provide predictive biomarkers for screening patients who benefit from NAC, and lay the foundation for exploring new therapeutic targets for BC. However, owing to high heterogeneity and insufficient precision of BC, the prediction for NAC response still remains a big challenge in BC management. Therefore, there is an urgent need to identify novel predictive molecular biomarkers that can further facilitate the selection of patients who are more likely to benefit from NAC.

Studies in bladder cancer, gastric cancer, ovarian cancer, and esophageal squamous cell carcinoma have shown that NAC can change the omics characteristics of tumor cells, which may further affect responses to subsequent therapy and patient prognosis [13–16]. In BC, similar studies have tended to focus on a single-level omics such as genomics or transcriptomics [17–19], rather than simultaneous multi-omics analyses, which are beneficial for a more comprehensive understanding of molecular changes in BC during NAC.

In the present study, we first established the genomic and transcriptomic profiles of breast tumors before and after treatment using a multi-omics characterization strategy that combined whole exome sequencing (WES) and RNA sequencing (RNA-seq) analyses. Molecular features related to NAC sensitivity were further analyzed by integrating omics and clinical characteristics, followed by confirmation assays of potential biomarkers using in vitro cell line models or clinical validation cohorts.

## Methods

### Patient population and samples

This study included four datasets: three datasets enrolled BC patients who received NAC (the NACBC sequencing set, the internal NACBC validation set, and the external Gene Expression Omnibus (GEO) validation set) and the fourth dataset enrolled BC patients who received adjuvant chemotherapy (the external The Cancer Genome Atlas (TCGA) validation set). Their characteristics are as follows.

**Fig. 1:**
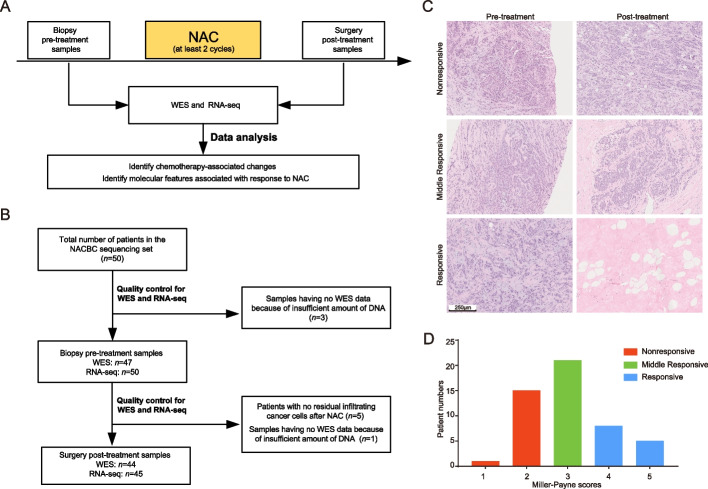
Study overview. **A** A schematic diagram of sample collection in the context of neoadjuvant chemotherapy (NAC), followed by whole exome sequencing (WES), RNA sequencing (RNA-seq), and data analyses. **B** The final number of samples in the NACBC sequencing set for analysis. All samples were acquired from 50 patients. In the pre-treatment group, there were 47 tumor samples for WES and 50 for RNA-seq. In the post-treatment group, there were 44 tumor samples for WES and 45 for RNA-seq. **C** Representative pathological images of tumors by hematoxylin–eosin staining from the responsive, middle responsive, and nonresponsive patients. Bar, 250 μm. **D** The distribution of patients with different Miller–Payne scores in the responsive, middle responsive, and nonresponsive groups

In the NACBC sequencing set, eligible patients diagnosed with primary BC were treated with NAC, followed by surgery at The Second Hospital of Shandong University between March 2013 and August 2019. The inclusion criteria were: (1) patients were newly diagnosed with histologically confirmed non-metastatic BCs; (2) patients received at least two cycles of NAC before surgery; (3) Biopsies samples before NAC, and surgical samples after NAC (if there was residual disease) could be collected (Fig. 1A). Pre-NAC samples were collected by biopsy. For the post-NAC samples collection, immediately after the residual disease was resected, the specimens were delivered to the Department of Pathology for gross and microscopic examination. Post-NAC samples were collected without compromising the surgical pathological evaluation of the resection specimen. The tissues were submerged in RNAlater or frozen directly in liquid nitrogen until further use. In this set, samples from 50 patients were used for WES and/or RNAseq. Forty-seven pre- and 44 post-treatment tumor samples and matched germline DNA samples were analyzed by WES. Fifty pre- and 45 post-treatment tumor samples were analyzed by RNA-seq (Fig. 1B, Additional file 1: Fig. S1). This sequencing set was used to identify molecular changes following NAC and screen for molecular features associated with response to NAC in BC.

After NAC, the abundance of residual tumor cells in the primary breast tumor bed site was evaluated according to the Miller–Payne (MP) histological grading system [20]. It was performed on all patients according to the standard criteria by two independent, blinded pathologists. Tumors were classified into three groups: the responsive was defined when infiltrating cancer cells were significantly reduced by > 90% with only small clusters or widely dispersed individual cancer cells, or there were no infiltrating cancer cells at the original tumor bed site (MP scores: 4 or 5); the middle responsive referred to those with a reduction of cancer cells between 30 and 90% (MP scores: 3); and the nonresponsive were those with a reduction of tumor cells between 0 and 30% (MP scores: 1 or 2) (Figs. 1C and D).

Clinicopathological characteristics including age at initial diagnosis, tumor histologic type, tumor size, lymph node status, histologic grade, neoadjuvant therapies, and estrogen receptor (ER), progesterone receptor (PR), human epidermal growth factor receptor 2 (HER2), and Ki-67 status were collected. ER and PR status were assessed using immunohistochemistry (IHC), with positivity defined as ≥ 1% of tumor cells being positive immunostaining [21]. HER2 status was assessed using IHC and in situ hybridization (ISH) analysis if necessary. Positive HER2 status was determined as an IHC score of 3 + (more than 10% tumor cells with intense, complete and homogeneous membrane staining of HER2) or a positive ISH result. Clinical tumor and lymph node stage before NAC were determined by an experienced physician through physical examination and ultrasonography by at least two independent radiologists.

In the internal NACBC validation set, a tissue microarray (TMA) was constructed from formalin-fixed paraffin-embedded pre-treatment biopsies from patients who were diagnosed with primary BC and treated with NAC at the same center as the NACBC sequencing cohort between January 2013 and December 2018. Invasive cancer sites in donor paraffin blocks were identified by an experienced pathologist using matching hematoxylin and eosin reference slides. Then, the TMA was constructed using 2 mm cores by a tissue microarray facility (3DHISTECH, Budapest, Hungary). After the TMA was fabricated, it was sectioned into 4-μm-thick tissue slices and stained with hematoxylin–eosin. The quality of the TMA was evaluated by two experienced pathologists. In the subsequent IHC analysis, cores without invasive carcinoma were excluded. The patient inclusion criteria were as follows: (1) with newly diagnosed, histologically confirmed non-metastatic BC; (2) received at least two cycles of NAC before surgery; (3) received a standard treatment (including surgery and chemotherapy); (4) with complete follow-up information available; (5) whose tumor tissue on the TMA being confirmed as invasive carcinoma by hematoxylin–eosin staining; and (6) ADGRA2 and ADRB3 expression could be assessed. A final population of 156 patients was included in the NACBC validation set. Clinicopathological assessments of this validation set were the same as for the NACBC sequencing set. This validation set was used to analyze the relationship between ADGRA2 or ADRB3 protein expression and pathological response and prognosis of patients.

In the GEO validation set, the GSE25066 dataset was used to study genomic predictors of response and survival following neoadjuvant taxane-anthracycline chemotherapy in BC [22]. The GSE25066 is a combination of GSE25055 and GSE25065 datasets. Gene expression data were log2 transformed and scaled to a reference distribution of 1,322 BC specific genes. The GSE25066 dataset included a total of 508 patients with complete DFS event information; however, whether 20 of the patients had residual disease after NAC was unknown. We extracted the expression level of *ADGRA2* and *ADRB3* for analysis with the cut-off values being determined by using the maximum Youden Index [23, 24]. This set was used to validate the relationship between *ADGRA2* or *ADRB3* mRNA expression and pathological response and prognosis of patients.

The TCGA validation set included 1,085 female BC patients. It was used to further validate the role of *ADGRA2* and *ADRB3* in chemotherapy response as did with the GSE25066 dataset. Therefore, only the 566 patients who received chemotherapy and had prognostic information available were analyzed for the mRNA expression in the present study. This validation set was used to analyze the relationship between *ADGRA2* or *ADRB3* mRNA expression and patient’s prognosis.

### Isolation of genomic DNA and RNA

Total DNA was isolated from fresh frozen tissue samples using the QIAamp DNA Mini Kit (Qiagen, Hilden, Germany), and blood samples using the QIAamp DNA Blood Mini Kit (Qiagen). TRIzol reagents (Tiangen, Beijing, China) was used to extract RNA from fresh frozen tumor tissue. The purity of total DNA and RNA were estimated by measuring the absorbances at 260 nm (A_260_) and 280 nm (A_280_) using a NanoPhotometer® spectrophotometer (IMPLEN, Munich, Germany). The extracted DNA and RNA were considered pure and suitable for subsequent experiments when the A_260_/A_280_ ratio was within the range of 1.8 to 2.0. A mass ≥ 3 µg was considered to meet the experimental requirements for sequencing sample library construction. RNA samples were also tested by formaldehyde denaturing gel electrophoresis, wherein the rRNA ratio (28S/18S) needed to be ≥ 1.5, otherwise it meant that the RNA had degraded.

### DNA sequencing

Qualified genomic DNA samples were prepared from tissue and peripheral blood samples for WES. Briefly, 3 μg of DNA was sheared into short fragments of 150 to 200 bp using an ultrasonicator Covaris M220 (Thermo Fisher Scientific, Waltham, MA, USA). Quality control was performed using a 2100 Bioanalyzer system (Agilent Technologies, Santa Clara, CA, USA) after fragmentation. The library was constructed using a KAPA Library Quantification kit (KAPA Biosystems, South Africa) and “SureSelectXT Human All Exon V6” (Agilent Technologies) according to the manufacturer’s protocol. The kit was used to enrich the 357,999 exons from the 21,522 genes, covering approximately 60 Mb of the human genome. Validated DNA libraries were sequenced with paired-end runs on an Illumina NovaSeq 6000 (Illumina Inc., San Diego, CA, USA) by the CapitalBio (Beijing, China).

### RNA sequencing

Library construction for RNA-seq was performed as described in the TruSeq RNA Sample Preparation Kit. Briefly, isolated total RNA was reverse-transcribed into cDNA with poly-dT primers using the Hifair® kit (Yeasen Biotech, Shanghai, China). The RNA-seq library was prepared by cDNA synthesis, end repair, 3′ adenylation, adaptor ligation, amplification, and product purification. Quality control was performed using the Agilent 2100 Bioanalyzer (Agilent Technologies) with a DNA chip. After quantification with a NanoPhotometer® spectrophotometer (IMPLEN), libraries were sequenced with paired-end runs on an Illumina NovaSeq 6000 (Illumina Inc.) by the CapitalBio.

### WES data analysis

The fastp (v0.20.0) [25] was used to filter raw data. The specific conditions were as follows: the adapter in the sequence was identified and cut off in the read with a minimum length of the reserve being 100 bp. If a read with > 5% “N” bases and/or > 50% low-quality base, the entire pair of reads were removed. Valid sequencing data were aligned to the human reference genome (GRCh38) using the Burrows–Wheeler Aligner (v0.6.1) [26], and the resulting BAM files were preprocessed using the Sentieon (v202010). Sequencing quality statistics were obtained using the fastp. The average target sequencing coverage depth of tumor and matched germline samples was approximately 100 × .

To identify all somatic variants in the samples, we used two pipelines (Sentieon TNseq and TNscope) [27] to detect for single nucleotide variants (SNVs) and indels, and matched normal samples were used to exclude germline variations. Somatic mutations were annotated using the ANNOVAR (v20160201) [28]. To obtain the accurate mutation call set, two caller consensus mutations were performed for additional filtering. The bcftools v1.10.2 [29] (https://github.com/samtools/bcftools) was used for further filtering to reduce false positive calls with the following criteria: (1) quality score ≥ 20; (2) FisherStrand ≤ 60.0; (3) StrandOddsRatio ≤ 3; (4) sequencing depth in the region ≥ 30; (5) sequence reads in support of the variant call ≥ 2; and (6) variant allele frequency (VAF) ≥ 0.05.

Based on the somatic mutation data, we conducted somatic mutation signature analysis using the deconstructSigs1.9.0 R package with the default parameters [30, 31]. The COSMIC signatures were used as the reference to annotate the identified signatures. The MuSiC2 was used to explore significantly mutated genes (false discovery rate [FDR] < 0.1) [32]. Tumor mutation burden was calculated by the Maftools R package [33]. When calculating tumor mutational burden and analyzing mutations related to chemotherapy sensitivity, only mutations with the following functional classifications were considered [34–36]: frame_shift_del, frame_shift_ins, in_frame_del, in_frame_ins, missense_mutation, nonsense_mutation, nonstop_mutation, splice_site, and translation_start_site. Somatic copy number alterations (SCNAs) were detected using the CNVkit [37], and genomic regions with significant amplifications or deletions in the samples were summarized by the GISTIC2.0 [38]. Tumor purity was estimated by the ABSOLUTE [39].

Germline variants were identified using the Sentieon Haplotyper tool [40]. The ClinVar database was used to annotate known pathogenic and likely pathogenic variants. The 28 cancer predisposition genes [41] were evaluated. They include 12 established breast cancer–predisposition genes (*ATM*, *BARD1*, *BRCA1*, *BRCA2*, *CDH1*, *CHEK2*, *NF1*, *PALB2*, *PTEN*, *RAD51C*, *RAD51D*, and *TP53*) and 16 candidate predisposition genes (*BLM*, *BRIP1*, *CDKN2A*, *ERCC3*, *FANCC*, *FANCM*, *MLH1*, *MRE11A*, *MSH2*, *MSH6*, *NBN*, *RAD50*, *RECQL*, *RINT1*, *SLX4*, and *XRCC2*).

### RNA-seq analysis

Raw data were filtered following standard pipelines, and reads that did not meet the analysis criteria were deleted by fastp. The HISAT2 [42] was then used to map the filtered data to the human reference genome (GRCh38). Finally, the FeatureCounts [43] and StringTie [44] were used to perform transcript reconstruction and statistics on the basis of the reads-reply results.

Differentially expressed genes between subgroups were identified using the DESeq2 R package [45]. For comparisons between pre- and post-treatment samples, we performed a paired analysis on the basis of patient IDs. The WebGestalt 2019 [46] was used for the gene set enrichment analysis [47]. Transcripts per million (TPM) was used to measure the expression levels of genes, and the composition of immune and stroma cells were calculated using the xCell [48].

### Cell culture and chemicals

The human BC cell line HCC1806 was purchased from the BeNa Culture Collection (Kunshan, China). BT-549, MDA-MB-231, MDA-MB-453, SK-BR-3, T47D, BT-474, and MCF-7 cells were purchased from the Zhong Qiao Xin Zhou Biotechnology Co. (Shanghai, China). HCC1806, MDA-MB-231, MDA-MB-453, T47D, and BT-474 cells were maintained in RPMI 1640 medium (Corning Inc., Corning, NY, USA) supplemented with 10% fetal bovine serum (FBS; ExCell Bio, Shanghai, China) and 1% penicillin and streptomycin (Solarbio, Beijing, China). MCF-7 cells were cultured in MEM medium (Corning Inc.) supplemented with 10% FBS, 1% penicillin and streptomycin, and 0.005 mg/mL bovine insulin (Solarbio). SK-BR-3 cells were cultured in McCoy’s 5a medium (Macgene, Beijing, China) supplemented with 10% FBS and 1% penicillin and streptomycin. BT-549 cells were cultured in RPMI 1640 medium supplemented with 10% FBS, 1% penicillin and streptomycin, and 0.023 IU/mL insulin (Beyotime, Shanghai, China). All cell lines were cultured at 37 °C in a humidified atmosphere containing 5% CO_2_. All cell lines were authenticated by the Shanghai Biowing Applied Biotechnology Co. Ltd. (China) using a short tandem repeat profiling analysis before conducting experiments. Assessments of mycoplasma contamination using the MycoBlue Mycoplasma Detector (Vazyme, Nanjing, China) were performed prior to performing experiments to confirm that the cells used for experiments were free of mycoplasma contamination.

### Cell infection

The CDKAL1 wild type and mutant (CDKAL1_*P409L*_) cDNAs were cloned into the pCDH-CMV-MCS-EF1-BSD vector. The CENPT wild type, and CDKAL1_*P409L*_ mutants (CENPT_*R122G*_, and CENPT_*P442L*_) cDNAs were cloned into the pLenti-C-Myc-DDK-IRES-Puro vector. These two lentiviral vectors were purchased from the BioSune Biotechnology Co. Ltd. (Shanghai, China). Viral particles were prepared by transfecting HEK293T cells with the constructed or control plasmids in combination with packaging vectors using Lipofectamine 3000 transfection reagents (Invitrogen, Waltham, MA, USA). The cell supernatant was collected at 48 and 72 h after transfection. After the supernatant was filtered through a 0.45-μm filter, it was ultracentrifuged at 11,000 × *g* for 3 h at 4 °C using an Optima XPN-80 ultracentrifuge (Beckman Coulter, Brea, CA, USA). After ultracentrifugation, virus pellets were resuspended in PBS. Finally, a concentrated virus solution (plus polybrene) was used to infect cells 48 h before selection with the appropriate antibiotic.

### Quantitative real-time PCR (qPCR)

Total RNA was prepared from cells using a TRIzol reagent (Invitrogen) and reverse-transcribed to cDNA using the HiScript®III RT SuperMix for qPCR (+ gDNA wiper) kit (Vazyme). Primers are listed as follows: 5′-CTGCTGCATCTCAGTGTGAC-3′ (forward) and 5′-TCCTCAGCGCACAGTCTTGA-3′ (reverse) for *CDKAL1*; 5′-GCCTCTTCCCTCACCAGATCC-3′ (forward) and 5′-CACAATGTTTGGAGGAGCCAG-3′ (reverse) for *CENPT*; 5′- CATGTACGTTGCTATCCAGGC-3′ (forward) and 5′- CTCCTTAATGTCACGCACGAT-3′ (reverse) for *ACTB*. qPCR was performed on a QuantStudio 5 Real-Time PCR Instrument (Thermo Fisher Scientific) using a 2 × Universal SYBR Green Fast qPCR Mix (ABclonal, Wuhan, China). *ACTB* was used as the internal control, and the relative expression of target genes was calculated using the 2^−ΔΔCt^ method.

### Protein extraction and western blot analysis

To obtain whole-cell protein extracts, cells were lysed with 1 × SDS-PAGE Sample Loading Buffer (Beyotime). The cell lysates were denatured for 5 min at 95 °C. Equal amounts of proteins from cell lysates were electrophoresed on SDS-PAGE and transferred to polyvinylidene difluoride membranes (Millipore, Burlington, MA, USA). After blocking with 5% non-fat milk, the membranes were incubated with the indicated primary antibodies overnight at 4 °C, and then with horseradish peroxidase (HRP)-labeled secondary antibody at room temperature for 1 h. The membranes were washed three times (5 min per wash) with Tris-buffered saline containing Tween-20 (TBST) before and after antibody incubations. Finally, chemiluminescent HRP substrate (Millipore) was added to the membranes, and immunoreactive bands were detected by a chemiluminescent imaging system (Tanon, Shanghai, China). All experiments were repeated at least three times. The primary and secondary antibodies used in this study were as follows: CDKAL1 (Cat# ab169531, AbCam, Cambridge, UK), CENPT (Cat# ab86595, AbCam), β-actin (Cat# AC026, ABclonal), and HRP-AffiniPure Goat Anti-Rabbit IgG (H + L) (Cat# 111–035-003, Jackson ImmunoResearch, West Grove, PA, USA).

### Cell proliferation assays

For proliferation assays, a CCK-8 cell counting kit (Dojindo, Kumamoto, Japan) was used to assay the cell viability. Infected cells were plated in 96-well plates with a final volume of 100 μL of growth medium and incubated overnight under 5% CO_2_ at 37 °C. Ten drug concentrations were freshly prepared according to the half-log dilution method (10,000-fold range, docetaxel: 0–1 μM, epirubicin: 0–20 μM). The cells were treated with different concentrations of docetaxel (MedChemExpress, Houston, TX, USA) and epirubicin (MedChemExpress) with five replicates per condition. After 48 h, the CCK-8 assay was performed by incubating cells with a CCK-8 reagent for 2 h at 37 °C, and measuring the absorbance at 450 nm with an Infinite 200 PRO plate reader (TECAN, Männedorf, Switzerland). These data were used to calculate the cell viability at different drug concentrations. The growth and dose inhibition curves were plotted and analyzed using the GraphPad Prism 8.3.0 (GraphPad Software, Inc., San Diego, CA, USA). The IC_50_ values were determined by nonlinear regression analysis of the plots of the percentage of growth inhibition vs. the log of inhibitor concentrations. All experiments were repeated at least three times, and data are expressed as mean ± SD.

#### IHC

In IHC analyses, the EnVision method was used to assess the expression of ADGRA2 and ADRB3. Briefly, the TMA was sectioned into 4-μm-thick tissue sections. After deparaffinization, rehydration, antigen retrieval using the PT Link for Pre-Treatment reagent (Agilent Technologies), and blockage of endogenous peroxidase activity, the sections were incubated with the rabbit anti-ADGRA2 (1:40; Cat# ab198817, Abcam) or rabbit anti-ADRB3 (1:50; Cat# ab140713, Abcam) antibodies for 1 h at room temperature, followed by incubation with a secondary antibody (Cat# SM802, DAKO, Glostrup, Denmark) for 20 min at room temperature. Negative controls only included the antibody dilution buffer (DAKO, Cat# DM830) without a primary antibody. The staining was assessed independently by two pathologists blinded to patient information. The IHC scoring was based on the proportion and intensity of positively stained invasive BC cells on slides. The proportion of positive tumor cells was recorded as a percentage. The intensity scores represent the average staining intensity of the positive tumor cells (negative = 0; weak staining = 1; moderate staining = 2; and strong staining = 3). The proportion and intensity scores were then multiplied to obtain a total IHC score, which ranges from 0 to 300. According to whether the patient had a DFS event (as a judgment standard), we analyzed the receiver operating characteristics (ROC) curve of ADGRA2 and ADRB3 expression. The maximum Youden Index was again used to determine the optimal cut-off value to divide patients into high and low expression groups. An IHC score ≥ 70 for ADGRA2 and ≥ 80 for ADGRA3 was considered high expression.

### Statistical analyses

The Student’s t-test and Wilcoxon test were used to compare continuous variables, while the Pearson’s chi-square test and Fisher’s exact test were used to compare unordered categorical variables. The log-rank test was used to compare differences in breast cancer-specific survival (BCSS) and DFS between patients with a high and low expression of *ADGRA2* and *ADRB3*. Cox regression models were used to estimate the HRs at 95% CIs for BCSS and DFS events associated with the expression of *ADGRA2* and *ADRB3*. Age and Ki-67 level were adjusted as continuous variables; menopausal status, endocrine therapy, radiotherapy, and other clinical factors (cT, cN, histological grade, ER status, PR status, HER2 status) were adjusted as categorical variables. All statistical analyses were performed using the R packages version 4.2.0 (https://cran.r-project.org/) and the SPSS version 23.0 (IBM, Armonk, NY, USA). *P* < 0.05 were considered statistically significant and *P* < 0.1 marginally significant.

## Results

### Characteristics of BC patients treated with NAC in the sequencing set

To investigate the genomic and transcriptomic features of tumors before and after NAC, we enrolled 50 BC patients who received NAC in the NACBC sequencing set for this study (Figs. 1A and B). The median age at diagnosis was 49 years (range: 27–68 years). The stages of BCs at diagnosis were stage I (*n* = 4), stage II (*n* = 41), and stage III (*n* = 5). ER, PR, and HER2 positive patients accounted for 82%, 56%, and 40% of the cohort, respectively. Among them, 82% (41/50) patients received a taxane-based regimen as first-line treatment (Additional file 2: Tables S1 and S2).

### Changes in somatic mutation and copy number variation between paired pre- and post-treatment tumors

**Fig. 2:**
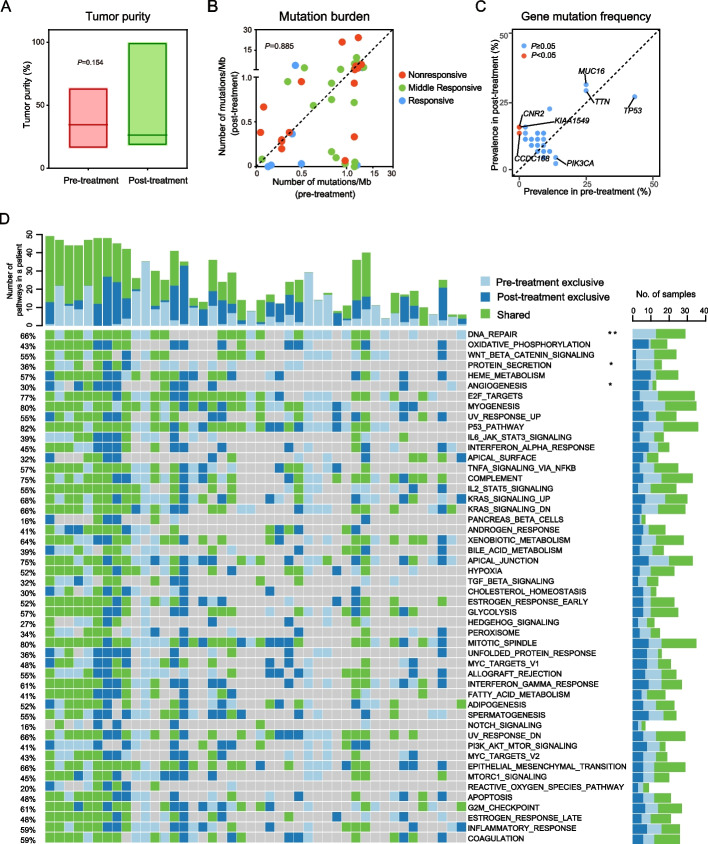
Changes in gene mutation, mutation burden, and the MSigDB pathway between the paired pre- and post-treatment tumor samples. Comparison of tumor purity (**A**) and mutation burden (**B**) between the 44 paired pre- and post-treatment tumors. *P* values are calculated based on the Wilcoxon signed-rank test. **C** The most frequently mutated genes before and after NAC. **D** Mutations associated with the MSigDB pathway in the pre- and post-treatment tumors. Bars on the top indicate the number of pathways affected in a given patient, and colored bars indicate if the variant was only found in the pre- or post-treatment tumors, or shared in both. *P* values in panels C and D are calculated based on the Pearson’s chi-square test; **P < 0.01, *P < 0.05

There were no statistical differences in tumor purity among the 44 paired pre- and post-treatment tumors (Fig. 2A). We further analyzed the somatic mutation and copy number variation (CNV) landscape changes between tumors in response to NAC. We identified 15,499 somatic SNVs (median: 139.5) and 598 somatic small indels (median: 4) in the pre-treatment tumors, and 27,458 nucleotide substitutions (median: 134) and 770 small indels (median: 5) in the post-treatment tumors. SNV analysis showed that C > T substitutions occurred more frequently than any other SNVs in all the tumors and that the fraction of transversion mutations (C > A) was reduced after NAC (*P* = 0.020, Additional file 1: Fig. S2). We examined the mutational signature weights among the Catalogue of Somatic Mutations in Cancer (COSMIC) signatures based on the frequency of 96 different possible trinucleotide substitutions. However, we did not detect any statistically significant changes in the COSMIC mutational signatures in the cohort (Additional file 1: Fig. S3, Additional file 2: Table S3).

In total, 4,433 and 6,767 nonsynonymous mutations were identified in the 44 paired pre- and post-treatment tumors, respectively. There were no statistically significant changes in mutation loads in the cohort (Fig. 2B), and the most frequently altered genes were *TP53*, *TTN*, and *MUC16* in both pre- and post-NAC samples although the change of these three genes was not statistically significant (Fig. 2C). However, compared with the pre-treatment tumors, *CNR2*, *KIAA1549*, and *CCDC168* gene mutations were solely observed in the post-treatment tumors under the pressure of chemotherapy (*P* < 0.05, Additional file 2: Table S4). We next performed gene set enrichment analyses on the Molecular Signatures Database (MSigDB) of hallmark gene sets and identified that three pathways were significantly affected by mutations. The mutation rates of the DNA REPAIR and PROTEIN SECRETION pathways significantly decreased in the post-treatment tumors. Of the 44 paired pre- and post-treatment tumors, 29 pre-treatment and 16 post-treatment tumors contained gene mutations in the DNA REPAIR pathway (*P* = 0.006, Fig. 2D) while there were 14 pre-treatment and 4 post-treatment tumors contained gene mutations in the PROTEIN SECRETION pathway (*P* = 0.042, Fig. 2D). Conversely, a higher mutation rate of ANGIOGENESIS pathway was observed in the post-treatment group (4 out of 44) than in the pre-treatment group (11 out of 44) (*P* = 0.047, Fig. 2D).

SCNA analyses identified 13 amplifications and 23 deletions in the pre-treatment tumors, and 13 amplifications and 18 deletions in the post-treatment tumors (Additional file 1: Fig. S4A). Of them, 4 amplifications (1q21.3, 11q13.3, 15q26.3, and 17p11.2) and 11 deletions (1p36.31, 1q44, 2q37.3, 3p14.1, 6p22.1, 6q27, 8p23.3, 11q12.1, 12p13.2, 13q34, and 15q13.3) only occurred in the pre-treatment tumors. In addition, 4 amplifications (2p11.2, 8q24.3, 11q13.4, and 11p15.4) and 6 deletions (1q43, 4q35.1, 5p15.33, 6p21.33, 6q22.33, and 16p13.11) were specifically detected in the post-treatment tumors (Additional file 1: Fig. S4B). The 4q35.1 region contains the *CENPU* gene, whose mRNA expression was also downregulated in post-treatment tumors (Additional file 1: Fig. S4C).

### Changes in gene expression and cell composition following NAC

**Fig. 3:**
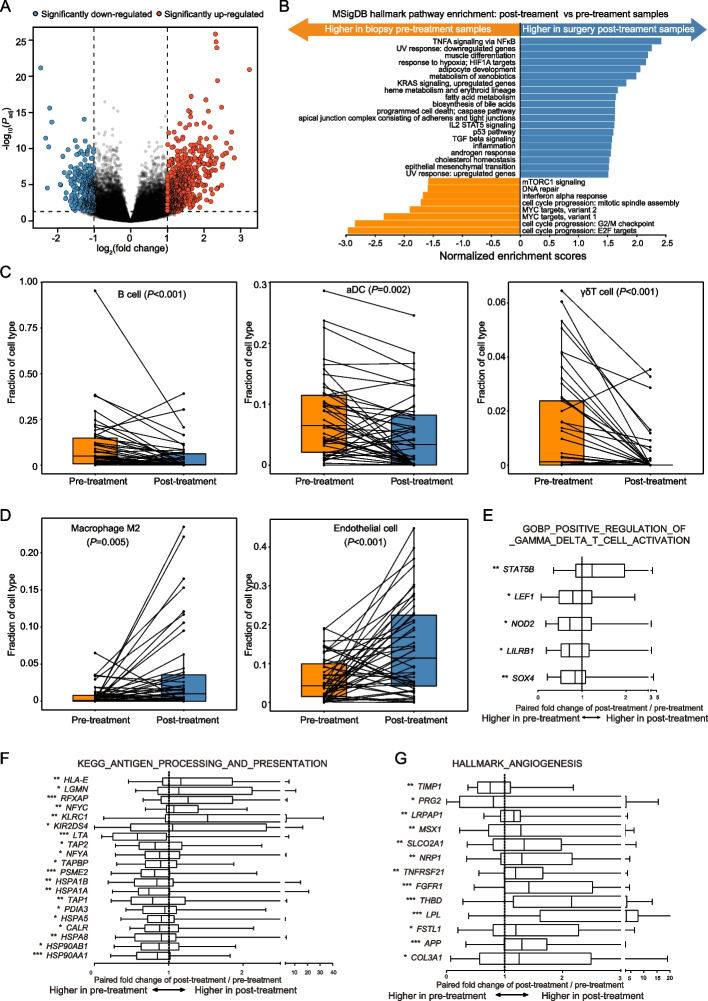
Changes in gene expression, tumor-infiltrating immune and stromal cell composition following NAC. **A** Volcano plots showing differentially expressed genes (DEGs) between the matched pre- and post-treatment tumors. Significant DEGs are shown as red (upregulated) and blue (downregulated) dots (fold change > 2, FDR < 0.05). **B** Significantly down­ and up-regulated pathways following NAC (FDR < 0.01). **C**, **D** The fractions of B cell, M2 macrophage, activated dendritic cell (aDC), endothelial cell, and gamma delta T (γδT) cell in the pre- and post-treatment tumors. *P* values are calculated based on the Wilcoxon signed-rank test. **E**–**G** The expression of DEGs was significantly related to positive regulations of γδT cell activation (**E**), antigen processing and presentation (**F**), and angiogenesis (**G**) between the pre- and post-treatment tumors. Values are presented as paired fold changes of post-/pre-treatment. *P* values were calculated by the Wilcoxon signed-rank test. ***P < 0.001, **P < 0.01, *P < 0.05

Differential gene expression analyses between the paired pre- and post-treatment tumors identified 1,130 differentially expressed genes (DEGs), including 705 upregulated and 425 downregulated genes (fold change > 2, FDR < 0.05, Fig. 3A). Compared with the pre-treatment tumors, gene enrichment analyses showed that gene sets associated with cell cycle progression (FDR < 0.001) and DNA repair were significantly downregulated (FDR = 0.007), whereas gene sets associated with response to hypoxia/HIF1A targets (FDR < 0.001) and KRAS signaling (FDR < 0.001) were upregulated in the post-treatment tumors (FDR < 0.01, Fig. 3B).

We further analyzed the cell composition of each tumor using the xCell algorithm [48] and compared the changes between the pre- and post-treatment tumors based on the Wilcoxon signed-rank test. The results indicated that after NAC, the fractions of B cells (*P* < 0.001), activated dendritic cells (aDCs, *P* = 0.002), and gamma delta T cells (γδT cells, *P* < 0.001) were decreased (Fig. 3C), whereas the fractions of M2 macrophages (*P* = 0.005), and endothelial cells (P < 0.001) were increased in the post-treatment tumors (Fig. 3D). We next compared the expression of immune checkpoint molecules and genes in antigen processing/presentation, positive regulation of γδT cell activation, and angiogenesis pathways in the pre- and post-treatment tumors. The results showed that compared with the pre-treatment tumors, the expression of *LAG3* gene was significantly decreased (*P* = 0.024), while the expression of *SIGLEC15* was significantly increased (*P* < 0.001) in the post-treatment tumors (Additional file 1: Figs. S5A and B). Marginal downregulation of *CTLA*4 (*P* = 0.05), *PD-L1* (*P* = 0.072), and *PD-1* (*P* = 0.067) genes were also observed in the post-treatment samples (Additional file 1: Figs. S5C-E). Most of the genes related to antigen processing and presentation (70%), and positive regulation of γδT cell activation (80%) were significantly downregulated (all *P* < 0.05, Figs. 3E and F; Additional file 2: Table S5), while 84.62% of genes related to the angiogenesis pathway were upregulated in the post-treatment tumors (all *P* < 0.05, Fig. 3G, Additional file 2: Table S5). We further analyzed the cell component fraction changes in subgroups with different degrees of NAC responses in the post-treatment tumors. A decrease in B cell fraction was observed in the middle responsive and nonresponsive groups, and a decrease in aDCs composition was only detected in the nonresponsive group (Additional file 1: Fig. S6).

### Somatic mutational analyses identify *CDKAL1*_*P409L*_ mutation decreases NAC sensitivity in BC

**Fig. 4:**
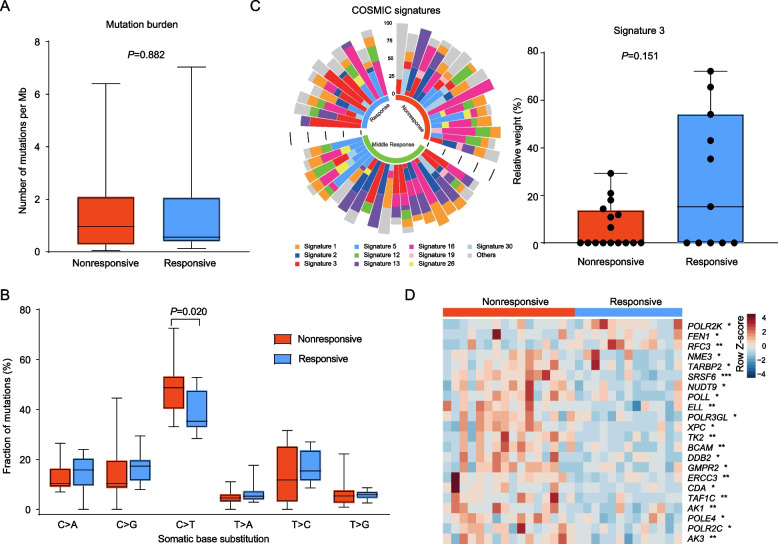
Mutation signatures in the pre-treatment tumors. Comparison of tumor mutation burden (**A**) and nucleotide substitutions (**B**) between the nonresponsive and responsive groups. **C** Distributions of the 10 main COSMIC signatures in the different NAC responsive groups across the 47 pre-treatment samples (*left*). Comparison of the relative weights of the signature 3 between the nonresponsive and responsive groups (*right*). **D** Heatmap comparison of the 22 genes statistically significantly related to the DNA repair pathway between the responsive and nonresponsive pre-treatment tumors. *P* values were calculated based on the Wilcoxon rank sum test. ***P < 0.001, **P < 0.01, *P < 0.05

To screen for molecular features related to NAC susceptibility, we compared the genomic differences between the nonresponsive (*n* = 16) and responsive (*n* = 11) groups of the pre-treatment tumors by analyzing the WES data. We observed no statistically significant differences in mutational loads between the two groups (Fig. 4A). Among the six possible base pair substitutions, the proportion of C > T substitutions was lower in the responsive group (39.35%) compared with the nonresponsive group (48.89%, *P* = 0.020, Fig. 4B), especially when the substitution site was flanked by C and G (responsive vs. nonresponsive: 3.93% vs. 6.87%, *P* = 0.022, Additional file 1: Fig. S7). Analyses of mutational signature weights for the COSMIC signatures demonstrated that a lower weight of signature 3, which is associated with failure of DNA double-strand break-repair by homologous recombination, in the nonresponsive group (range: 0%–29%) compared with the responsive group (range: 0%–72%); however, the difference was not statistically significant (*P* = 0.151, Fig. 4C). We further compared the differences in the expression of DNA repair pathway-related genes between the nonresponsive and responsive groups by analyzing the RNA-seq data. The expression of most DNA repair related genes was significantly upregulated in the nonresponsive group (all *P* < 0.05, Fig. 4D, Additional file 2: Table S6).

**Fig. 5:**
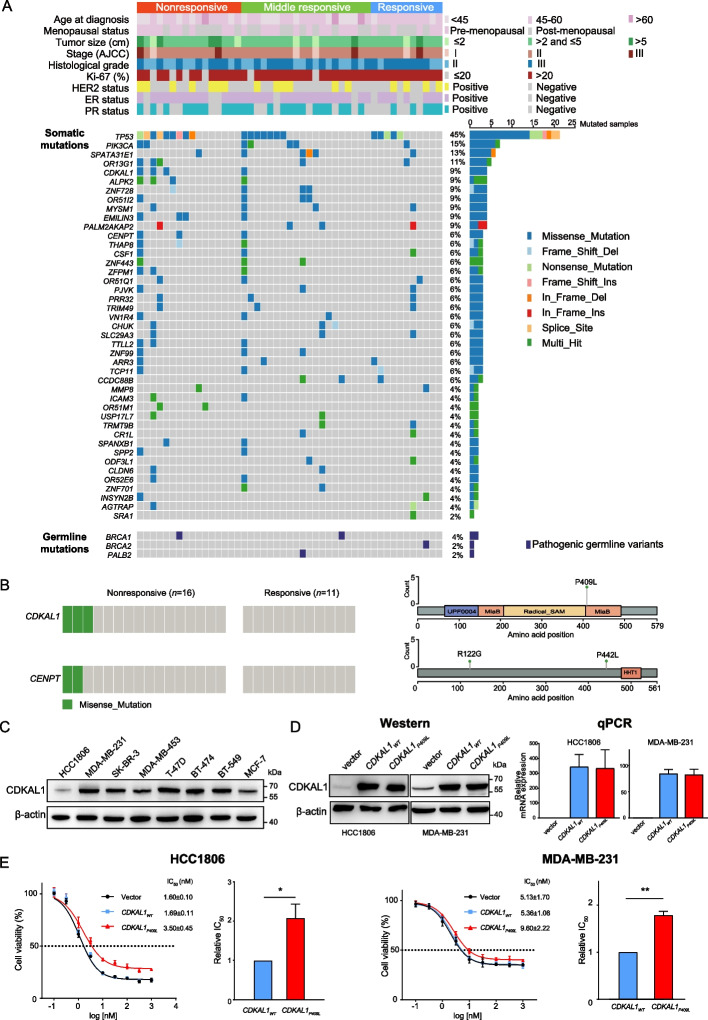
*CDKAL1*_*P409L*_ mutation decreased the sensitivity of cancer cells to docetaxel treatment. **A** Somatic and germline mutations in the 47 pre-treatment tumors and matched germline DNA. Samples were annotated for clinicopathological and molecular features (*top panel*). The types of somatic (*middle panel*) and germline (*bottom panel*) mutations of the indicated genes for each sample are displayed with colored squares. The histograms on the right-hand side show the accumulated number of alterations among the SMGs identified by the MuSiC2 (FDR < 0.1) or the pathogenic germline mutations classified in the ClinVar database. *AJCC*, The American Joint Committee on Cancer. **B** The distribution of potentially deleterious mutations in *CDKAL1* and *CENPT* in the nonresponsive and responsive pre-treatment groups (*left*). Diagrams representing the protein domains of potentially deleterious mutations (*right*). The “lollipopPlots” were generated using the maftools R package and manually edited. **C** The CDKAL1 expression in different human breast cancer cell lines as indicated was examined by western blot. **D** The expression of *CDKAL1*_*WT*_ and *CDKAL1P*_*409L*_ in HCC1806 and MDA-MB-231 cells infected with empty vector, *CDKAL1*_*WT*_ and *CDKAL1*_*P409L*_ lentiviruses by western blot and quantitative real-time PCR (qPCR) analyses. **E** IC_50_ assays of docetaxel. The proliferation of HCC1806 and MDA-MB-231 cells as described in (**D**) were determined with a CCK-8 cell counting kit at an increasing dose of docetaxel as indicated. The significance of relative IC_50_ values between *CDKAL1*_*WT*_ and *CDKAL1*_*P409L*_ cells with that of *CDKAL1*_*WT*_ cells as 1.0 were analyzed by paired t-test. Data represent mean ± SD (*n* = 3). *, *P* < 0.05; **, *P* < 0.01

As germline mutations may affect pathological response, we analyzed the presence of pathogenic germline variants of the 28 cancer predisposition genes [41]. We detected mutations in three of them in the NACBC sequence set (Fig. 5A): one *BRCA1* variant in the nonresponsive group, one *BRCA2* variant in the responsive group, and one *BRCA1* variant and one *PALB2* variant in the middle responsive group. No other pathogenic germline mutations were detected in the studied cohort. We found that the frequency of germline gene mutations did not differ between the nonresponsive (1 out of 16) and responsive (1 out of 11) groups (*P* = 1.0). Moreover, NAC sensitivity analyses including/excluding the patients carrying the aforementioned deleterious germline mutations did not alter the significance of the changes in the mutational signatures and the expression levels of DNA damage repair pathways (Fig. 4, Additional file 1: Figs. S7 and S8). Therefore, the pathological response observed in the sequencing set was not likely driven by the germline mutations in the breast cancer susceptibility genes.

We next analyzed the WES data using the MuSiC2 [32] and identified 43 significantly mutated genes (SMGs) in the 47 pre-treatment tumors (FDR < 0.1, Fig. 5A, Additional file 2: Table S7). To identify mutated genes that are associated with chemosensitivity in BC, we compared the differentially mutated genes in the nonresponsive and responsive groups. Mutations in *CDKAL1*, *ALPK2*, *EMILIN3*, *CENPT*, *OR51M1*, *THAP8*, *TTLL2*, and *ZFPM1* genes were primarily detected in the nonresponsive group but not in the responsive group. These mutations occurred in at least 2 nonresponsive tumor samples (Additional file 2: Table S8). We further conducted SIFT 4G [49] and PROVEAN [50] analyses to predict whether these gene mutational variations affect protein functions. The *CDKAL1* missense variant P409L (p.Pro409Leu, c.1226 C > T) and the *CENPT* missense variants R122G (p.Arg122Gly, c.364 A > G) and P442L (p.Pro442Leu, c.1325 C > T) were predicted to have a “deleterious” functional impact. These potentially deleterious mutations were also observed in at least 2 nonresponsive tumor samples (Fig. 5B, Additional file 2: Table S9).

We subsequently conducted in vitro studies to validate the effects of the deleterious *CDKAL1* and *CENPT* mutations on the responsiveness of BC cells to chemotherapeutics. We examined the mutations of both *CDKAL1* and *CENPT* in different BC cell lines using the Cancer Cell Line Encyclopedia (CCLE) online database [51]. All the cell lines tested did not harbor non-synonymous mutations except that MDA-MB-453 and BT-474 cells had nonsense and missense mutations of *CENPT*, respectively (Additional file 1: Fig. S9A). Western blots demonstrated that CDKAL1 and CENPT proteins were expressed in all cell lines tested (Fig. 5C, Additional file 1: Fig. S9B). HCC1806 and MDA-MB-231 for *CDKAL1*, and MDA-MB-231 and BT-549 cells for *CENPT* were selected for gene overexpression studies. Cells were infected with the lentiviruses that overexpress the wild type or mutants of the targeted genes *CDKAL1* (*CDKAL1*_*WT*_ and *CDKAL1*_*P409L*_), or *CENPT* (*CENPT*_*WT*_, *CENPT*_*R122G*_ and *CENPT*_*P442L*_). The efficiency of gene expression in infected BC cells was confirmed by western blot and real-time PCR (Fig. 5D, Additional file 1: Fig. S9C). The genotypes of the infected cells were also verified by Sanger sequencing (Additional file 1: Fig. S9D). The sensitivity of infected BC cells to chemotherapy drugs were determined with CCK-8 assays. The results indicated that HCC1806 cells overexpressing the p.Pro409Leu *CDKAL1* variant (*CDKAL1*_*P409L*_) decreased the sensitivity to docetaxel compared with cells overexpressing the wild type *CDKAL1* (*CDKAL1*_*WT*_), with the IC_50_ of docetaxel for *CDKAL1*_*P409L*_ and *CDKAL1*_*WT*_ cells being 3.50 ± 0.45 nM and 1.69 ± 0.11 nM, respectively (*P* < 0.05, Fig. 5E). A similar result was observed in MDA-MB-231 cells despite a higher expression of endogenous *CDKAL1*_*WT*_ compared with HCC1806 cells (*P* < 0.01, Fig. 5E). However, *CENPT* mutations did not affect the IC_50_ values of docetaxel in both MDA-MB-231 and BT-549 cells, and neither *CDKAL1* nor *CENPT* mutations affected the sensitivity of BC cells to epirubicin in the cell lines tested (Additional file 1: Fig. S9E).

To delineate the in-depth mechanism underlying these findings, we further compared RNA-seq data from the *CDKAL1*_*P409L*_ mutant and *CDKAL1*_*WT*_ wild type groups. The results showed that the HALLMARK_APOPTOSIS set was significantly enriched in the *CDKAL1*_*WT*_ wild type group compared with the *CDKAL1*_*P409L*_ mutant group (Additional file 1: Fig. S10A). The expression of anti-apoptosis gene *BCL2L2* was significantly higher, while the expression of pro-apoptosis genes *BAX* and *BID* were lower in the *CDKAL1*_*P409L*_ mutant group (Additional file 1: Fig. S10B). These data suggested that the *CDKAL1*_*P409L*_ mutation induced docetaxel resistance possibly by inhibiting apoptosis in BC cells.

### SCNA analyses demonstrate ADRB3 or ADGRA2 amplification induces worse NAC response and BC prognosis

**Fig. 6:**
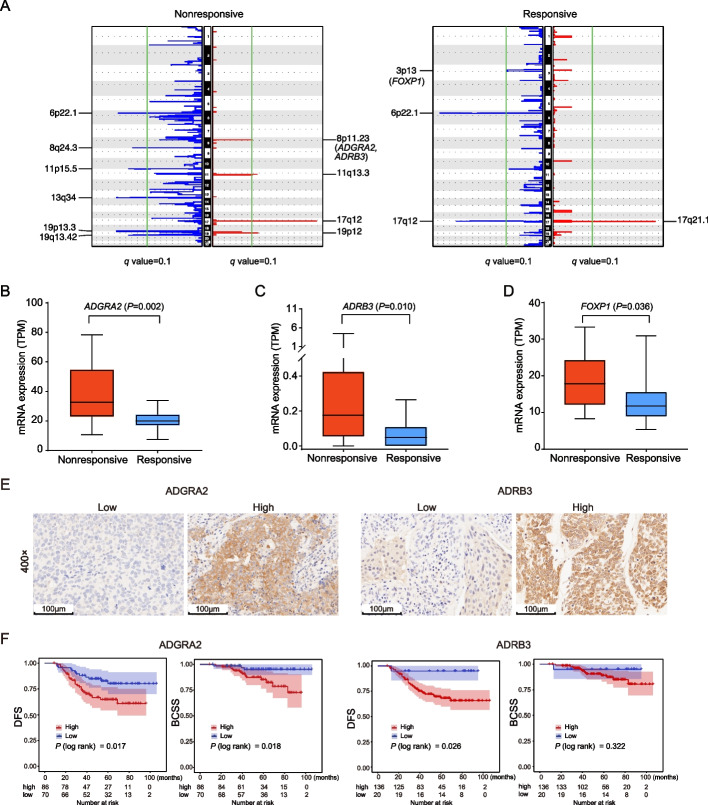
High ADGRA2 or ADRB3 expression is associated with worse NAC response and prognosis of BC patients. **A** The SCNA signal profiles identified by the GISTIC2.0 in the nonresponsive and responsive pre-treatment tumors. The significantly altered chromosome regions (q < 0.01) and the gene loci (*ADGRA2*, *ADRB3* and *FOXP1*) are annotated. The mRNA expression level of *ADGRA2* (**B**), *ADRB3* (**C**), and *FOXP1* (**D**) in the nonresponsive and responsive pre-treatment tumors from the RNA-seq data were shown as transcripts per million (TPM). **E** Representative immunohistochemistry staining of tumors with low and high expression of ADGRA2 and ADRB3 in the NACBC validation set (*n* = 156). Magnification: 400 × ; Bar, 100 μm. **F** Kaplan–Meier analyses of the DFS and BCSS in the NACBC validation set. Patients were stratified as high and low protein expression of ADGRA2 and ADRB3. *P* values were calculated based on the log-rank test

We conducted SCNA analyses of the paired tumor and normal samples for copy number amplification or deletion peaks between the genomes of nonresponsive and responsive pre-treatment samples using the GISTIC2.0 (FDR < 0.1). A unique amplification peak at 8p11.23 was identified in the nonresponsive group, which contained *ADGRA2* and *ADRB3* genes. Additionally, a unique deletion peak at 3p13 was observed in the responsive tumors, which contains the cancer related gene *FOXP1* (Fig. 6A).

We next examined whether changes in gene copy number affected the mRNA expression of *ADGRA2*, *ADRB3*, and *FOXP1* by analyzing the RNA-seq data in the pre-treatment tumors. The results indicated that the mRNA levels of *ADGRA2*, *ADRB3*, and *FOXP1* were significantly downregulated in the responsive group (*P* < 0.05, Figs. 6B-D). This was consistent with the SCNA analyses above.

A previous study has revealed that cytoplasmic FOXP1 expression in BC is associated with worse outcomes [52]. It is in agreement with our observation that *FOXP1* containing 3p13 region was deleted in the NAC-sensitive tumors. To validate the role of ADGRA2 and ADRB3 expression in chemotherapy response and prognosis, we used a NACBC validation set, which consisted of 156 pre-treatment tumor samples of BC patients who received NAC with follow-up information available. The baseline characteristics of this validation set are shown in Additional file 2: Table S1. ADGRA2 and ADRB3 protein expressions were examined by IHC on TMAs. The samples were divided into low and high expression groups based on the staining scores of ADGRA2 or ADRB3 (Fig. 6E). We demonstrated that a lower ADRB3 expression was significantly associated with a higher breast-only pCR rate (*P* = 0.031); no statistically significant correlations between ADGRA2 protein expression and baseline characteristics were found (Additional file 2: Table S10). Similar results were observed in an external GEO dataset [22], in which a lower *ADRB3* expression was correlated with a higher pCR rate, albeit with only marginal significance (*P* = 0.075). However, a significant association between a lower *ADGRA2* expression and a higher pCR rate was observed in this dataset (*P* = 0.047, Additional file 1: Fig. S11A).

We next determined whether ADGRA2 and ADRB3 expressions were associated with survival in our internal NACBC validation set, the external GEO and TCGA validation sets. In our NACBC validation set, Kaplan–Meier survival curve analysis showed that higher (versus lower) ADGRA2 protein levels were associated with a significantly reduced probability of DFS and BCSS (*P* = 0.017 and *P* = 0.018, respectively, Fig. 6F), and a high-level ADRB3 expression was associated with poor DFS (*P* = 0.026). In the GEO and TCGA validation sets, a lower *ADGRA2* expression was significantly associated with better prognosis (*P* < 0.05, Additional file 1: Figs. S11B and C). In the multivariate Cox proportional hazards regression model, after adjusting for age at diagnosis, clinical characteristics, and treatment, a higher ADGRA2 expression in BC cells significantly increased the risk of BCSS (hazard ratio [HR]: 8.042, 95% confidence interval [CI]: 1.874–35.012, *P* = 0.005) and DFS (HR: 2.487, 95%CI: 1.193–5.183, *P* = 0.015) events in the NACBC validation set. However, ADRB3 expression levels were not associated with BCSS (HR: 1.49, 95%CI: 0.138–16.112, *P* = 0.742) and DFS (HR: 3.36, 95%CI: 0.434–26.032, *P* = 0.246). These findings suggested that the expression of ADGRA2 and/or ADRB3 may be potential biomarkers for predicting the NAC response and the outcomes of BC patients.

## Discussion

Through combination analyses of both the WES and RNA-seq data, we first evaluated the differences in gene mutations, CNVs, gene expression, signaling pathways, and cellular components between tumors before and after treatment in primary BC, then examined the key molecular features related to NAC sensitivity of BC, and successfully identified *CDKAL1*_*P409L*_, ADGRA2 and ADRB3 as novel biomarkers for the selection of patients for NAC. These findings may help develop personalized treatments for BC.

In our cohort, the most frequently altered genes were *TP53*, *TTN*, and *MUC16* in both paired pre- and post-treatment tumors of BC. Following NAC, we observed acquired genetic alterations in *CNR2*, *KIAA1549*, and *CCDC168*. We further analyzed the functional biological processes or pathways that the mutated genes may affect. The mutation rate of the DNA repair pathway was significantly decreased after NAC, together with an expression downregulation of this pathway-related genes. The exact mechanism of how NAC affects the DNA repair-related genes via mutations or expression remains to be further investigated.

Our SCNA analysis demonstrated that *CENPU* was deleted in the post-treatment tumor samples in our cohort and that pathways related to cell cycle progression were downregulated in the RNA-seq data. *CENPU* has been shown to promote cell proliferation in various tumors [53–55], and previous studies have also found that tumors with a rapid growth rate are more sensitive to chemotherapy [56, 57]. Therefore, tumor cells with rapid proliferation are more likely to be eliminated by chemotherapy, while those with slow proliferation are more likely to remain. Our finding is in agreement with these studies in that cell cycle progression pathways were downregulated following NAC.

Cancer immunotherapy has achieved remarkable successes in certain molecular subtypes of BC patients [58]. The presence of immune cells and specific molecular expression patterns in the tumor microenvironment (TME) may affect the effectiveness of immunotherapy. The nature and composition of TME can vary over time with chemotherapy. The SWOG S0800 neoadjuvant trial showed no changes in tumor-infiltrating lymphocyte counts or *PD-L1* expression levels in residual disease (RD) cases [59]. However, another study found that both stromal tumor-infiltrating lymphocytes and CD8^+^ T cells were both decreased, while the expression of M2 macrophage-specific genes was significantly increased after treatment [17]. Therefore, the reported results from different centers are inconsistent. In our study, we found that NAC affected TME. NAC altered not only the expression levels of immune-related genes in BC tumor tissues, but also the composition of immune and stromal cells, including B cells, M2 macrophages, aDCs, endothelial cells, and γδT cells. DCs [60] and B cells [61] are professional antigen-presenting cells (APCs) of the immune system that can efficiently generate immune responses against tumors, including effective activation and expansion of CD8^+^ cytotoxic T lymphocytes that can specifically kill cancer cells [62–64]. In our study, we demonstrated that patients in the nonresponsive group displayed reduced levels of aDCs and B cells in the TME after NAC, suggesting that NAC may further induce insensitivity to immunotherapy in nonresponsive patients. Therefore, more attention should be paid to this patient population, especially when using immunotherapy drugs. In this group, the timing of immunotherapy and chemotherapeutic drugs should be carefully considered.

We also analyzed the molecular features associated with NAC response in the pre-treatment tumors. Different mutational processes often generate different combinations of single-nucleotide alterations, termed “signatures” [65]. The pattern of mutation signatures is associated with tumor sensitivity to chemotherapy and prognosis [13, 15]. In our cohort, there was a trend for higher levels of mutational signature 3 in the responsive group compared with the nonresponsive group. The signature 3 is associated with a failure of DNA double-strand break-repair by homologous recombination (https://cancer.sanger.ac.uk/signatures/signatures_v2/). Our RNA-seq data confirmed that most of the genes related to the DNA repair pathway exhibited higher expression levels in the nonresponsive tumors than in the responsive tumors, suggesting that the nonresponsive tumors may have a stronger ability to repair DNA damage, which is conducive to the survival of tumor cells. This finding is consistent with a previous study that found a higher proportion of signature 3 was associated with a higher rate of pCR after NAC [19]. Collectively, these results indicate that DNA repair deficiency confers increased chemotherapy sensitivity in BC.

We have identified a *CDKAL1* mutation in the nonresponsive group. Using in vitro studies, we demonstrated that BC cells with the *CDKAL1*_*P409L*_ mutation were more resistant to docetaxel. *CDKAL1* is a mammalian methylthiotransferase that biosynthesizes 2-methylthio-N6-threonylcarbamoyladenosine (ms2t6A) in tRNALys^(UUU)^ for the accurate translation of AAA and AAG codons [66]. Previous studies have shown that single nucleotide polymorphisms of *CDKAL1* are associated with susceptibility to and mortality from BC [67–69]. A germline genome-wide association study revealed that rs7453577 (located within *CDKAL1*) increased the pCR rate of NAC in HER2-negative BC patients who received bevacizumab [70]. However, to our knowledge, no studies have reported a relationship between *CDKAL1*_*P409L*_ and chemotherapy response. Our analysis of the RNA-seq data showed that the HALLMARK_APOPTOSIS gene set was significantly enriched in the *CDKAL1*_*WT*_ tumors compared with the *CDKAL1*_*P409L*_ tumors, resulting in a lower expression of the pro-apoptosis genes *BAX* and *BID*. Previous studies indicated that *CDKAL1* deficiency could induce the misreading of Lys codons and affect the synthesis of downstream proteins [71, 72]. We speculate that the *CDKAL1*_*P409L*_ mutation may decrease the ms2t6A modification of tRNA^Lys^ and downstream translation of pro-apoptotic proteins, thereby rendering mutant cells insensitive to docetaxel. Collectively, these results indicate that *CDKAL1*_*P409L*_ could be a biomarker for predicting insensitivity to NAC.

In the overall CNV analysis of the pre-treatment tumors, we found a gene amplification peak at 8p11.23 only in the nonresponsive subgroup. This chromosome region contains two genes *ADGRA2* and *ADRB3*, whose mRNA expression was higher in the nonresponsive group than in the responsive group, as shown in the RNA-seq analysis. *ADGRA2*, also known as *GPR124*, is an important member of the adhesion-type G protein-coupled receptor (aGPCR) family. *ADGRA2* was originally identified in the endothelial cells that form the neovasculature in invasive colorectal tumors [73]. Aberrant expression of *ADGRA2* has also been found in other types of cancers. In glioblastoma, it affected cancer cell proliferation by regulating the duration of mitotic progression [74]. In osteosarcoma, combination of β-elemene and paclitaxel inhibited bone neoplasm growth via downregulating *ADGRA2*, suggesting a potential role for *ADGRA2* in therapy response [75]. *ADRB3* has been proven to be a poor prognostic factor that accelerates cell proliferation in a variety of human cancers [76–78]. Additionally, blocking *ADRB3* promoted apoptosis and reduced chemoresistance in leukemia cells [79]. However, the role of *ADGRA2* and *ADRB3* in NAC response in BC has not been previously reported. In our study, we observed that low level expressions of *ADGRA2* or *ADRB3* increased the pCR rate in the NACBC validation and GEO sets, suggesting a negative correlation between *ADGRA2* or *ADRB3* amplification and NAC response in BC. Further survival analyses of all three datasets—the NACBC validation, GEO, and TCGA datasets—identified that a higher *ADGRA2* expression significantly increased risks of BCSS and DFS events, and a higher ADRB3 expression was associated with poorer DFS in the NACBC validation set. These findings suggest that *ADGRA2* or *ADRB3* amplification could predict worse NAC responses and poor outcomes in BC patients.

It is worth noting that our study is a single-center multi-omics analysis of BC before and after NAC. Conducting both genomic and transcriptomic studies in the same cohort has advantages to exploring the underlying mechanisms of genomic abnormalities. The consequence of any genomic abnormalities can be examined at a functional level. However, we also acknowledge that this study has limitations. Firstly, our study may suffer from potential biases introduced by the non-stratified population of molecular subtypes, such as ER, HER2 positive or triple negative, due to the relatively small sample size, which limited the power of our analyses. Secondly, although we conducted a series of in vitro studies and external dataset validations to confirm the key molecular features identified in the sequencing set, in vivo studies in animal models can be exploited next to provide further evidence. Therefore, future studies may focus on specific breast cancer subtypes with a big sample size for stronger evidence. A validation study using independent cohorts in other centers, perhaps on different ethnical populations, should also be considered.

## Conclusions

In summary, our study has revealed the dynamic genomic and transcriptomic landscape before and after NAC in BC, and identified multi-omics molecular signatures and potential biomarkers associated with NAC responsiveness and prognosis that can be used to make informed therapeutic decisions or serve as potential therapeutic targets in this population.

### Supplementary Information

**Additional file 1: Fig. S1.** Sample information of the NACBC sequencing set. **Fig. S2.** Comparison of different types of base substitutions in the pre- and post-treatment tumors. **Fig. S3.** Distributions of the ten main COSMIC signatures in each pre- and post-treatment tumor. **Fig. S4.** Copy number alteration between the pre- and post-treatment tumors. **Fig. S5.** Changes in immune related gene expression between the pre- and post-treatment tumors. **Fig. S6.** Changes in the composition of immune and stroma cells between the pre- and post-treatment tumors in different NAC responsive subgroups. **Fig. S7.** Comparison of 96 base substitution classifications in the pre-treatment tumors of responsive and nonresponsive groups. **Fig. S8.** Mutational signatures in the pre-treatment tumors with samples containing germline mutations removed. **Fig. S9.** Sensitivity of CDKAL1 and CENPT mutation to chemotherapy drugs in BC cells. **Fig. S10.** RNA-seq data analysis between the *CDKAL1*_WT_ and *CDKAL1*_P409L_ tumors. **Fig. S11.** Associations between *ADGRA2* or *ADRB3* expression and pCR or prognosis of BC patients. 

**Additional file 2: Table S1.** The summary of clinical information and sequencing data of the NACBC sequencing set. **Table S2.** Characteristics of patients included in the NACBC sequencing and validation sets. **Table S3.** The relative weights of the COSMIC mutational signatures. **Table S4.** Changed somatic variants in CNR2, KIAA1549, or CCDC168 after NAC. **Table S5.** Differences in the expression levels of genes related to KEGG_ANTIGEN_PROCESSING_AND_PRESENTATION, GOBP_POSITIVE_REGULATION_OF_GAMMA_DELTA_T_CELL_ACTIVATION, and HALLMARK_ANGIOGENESIS pathway between pre- and post-treatment. **Table S6.** Differences in the expression levels of genes related to HALLMARK_DNA_REPAIR pathway between the nonresponsive and responsive groups. **Table S7.** Significantly mutated genes in the pre-treatment tumors. **Table S8.** The frequency of non-synonymous SMG between the nonresponsive and responsive tumors.**Table S9.** Prediction of biological functional impact of somatic variants on protein functions. **Table S10.** Association of ADGRA2 or ADRB3 protein expression with clinicopathological parameters in the NACBC validation set.

## Data Availability

The WES and RNA-seq data of The NACBC sequencing datasets have been deposited in the Genome Sequence Archive [80] in the National Genomics Data Center [81], the Beijing Institute of Genomics (China National Center for Bioinformation), the Chinese Academy of Sciences (https://ngdc.cncb.ac.cn/gsa-human/browse/HRA003759) [82], and the processed datasets generated are deposited in the OMIX (https://ngdc.cncb.ac.cn/omix/release/OMIX002785) [83]. Based on the Regulation of the People's Republic of China on the Administration of Human Genetic Resources, the Human Genetic Resource Data are accessed via an application to the Data Access Committee for research purposes. Potential users need to complete and be approved for a data access request and then the data will be made available upon reasonable request according to the terms of the consent and the data use limitations for the subjects.

## Abbreviations

aDC: Activated dendritic cell
aGPCR: Adhesion-type G protein-coupled receptor
AJCC: The American joint committee on cancer
APC: Antigen-presenting cell
BC: Breast cancer
BCSS: Breast cancer-specific survival
CCLE: The cancer cell line encyclopedia
CI: Confidence interval
CNV: Copy number variation
COSMIC: The catalogue of somatic mutations in cancer
DEG: Differentially expressed gene
DFS: Disease-free survival
ER: Estrogen receptor
FBS: Fetal bovine serum
FDR: False discovery rate
GEO: The Gene Expression Omnibus
γδT cell: Gamma delta T cell
HER2: Human epidermal growth factor receptor 2
HR: Hazard ratio
HRP: Horseradish peroxidase
IHC: Immunohistochemistry
ISH: In situ hybridization
MP: Miller–Payne
MSigDB: The molecular signatures database
ms2t6A: 2-methylthio-N6-threonylcarbamoyladenosine
NAC: Neoadjuvant chemotherapy
OS: Overall survival
pCR: Pathologic complete response
PR: Progesterone receptor
qPCR: Quantitative real-time PCR
RD: Residual disease
RNA-seq: RNA sequencing
ROC: Receiver operating characteristics
SCNA: Somatic copy-number alteration
SMG: Significantly mutated gene
SNV: Single nucleotide variant
TBST: Tris-buffered saline containing Tween-20
TCGA: The Cancer Genome Atlas
TMA: Tissue microarray
TME: Tumor microenvironment
TPM: Transcripts per million
VAF: Variant allele frequency
WES: Whole exome sequencing
