# Supplementary material for: Genomic and transcriptomic analysis of breast cancer identifies novel signatures associated with response to neoadjuvant chemotherapy
Source: Genome Med. 2024 Jan 12;16:11. doi: 10.1186/s13073-024-01286-8 (PMC10787499; doi:10.1186/s13073-024-01286-8)
Supplement: Supplementary file 1 — Additional file 1: Fig. S1. Sample information of the NACBC sequencing set. Fig. S2. Comparison of different types of base substitutions in the pre- and post-treatment tumors. Fig. S3. Distributions of the ten main COSMIC signatures in each pre- and post-treatment tumor. Fig. S4. Copy number alteration between the pre- and post-treatment tumors. Fig. S5. Changes in immune related gene expression between the pre- and post-treatment tumors. Fig. S6. Changes in the composition of immune and stroma cells between the pre- and post-treatment tumors in different NAC responsive subgroups. Fig. S7. Comparison of 96 base substitution classifications in the pre-treatment tumors of responsive and nonresponsive groups. Fig. S8. Mutational signatures in the pre-treatment tumors with samples containing germline mutations removed. Fig. S9. Sensitivity of CDKAL1 and CENPT mutation to chemotherapy drugs in BC cells. Fig. S10. RNA-seq data analysis between the CDKAL1WT and CDKAL1P409L tumors. Fig. S11. Associations between ADGRA2 or ADRB3 expression and pCR or prognosis of BC patients. [file 13073_2024_1286_MOESM1_ESM.pdf]

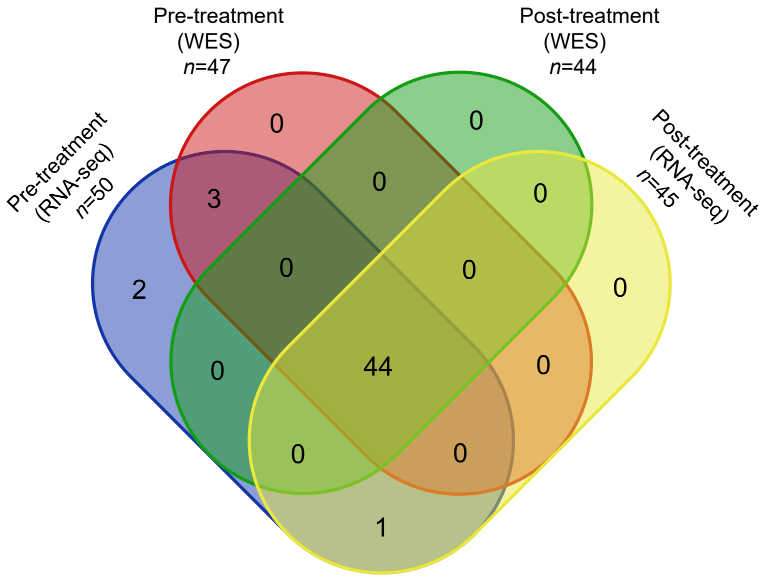

Fig. S1. Sample information of the study cohort. The Venn diagram of the NACBC sequencing samples (based on 50 BC cases who received NAC before surgery). Among them, 44 patients were available for the paired WES, and 45 patients were available for the paired RNA-seq.

Somatic base substitution summary

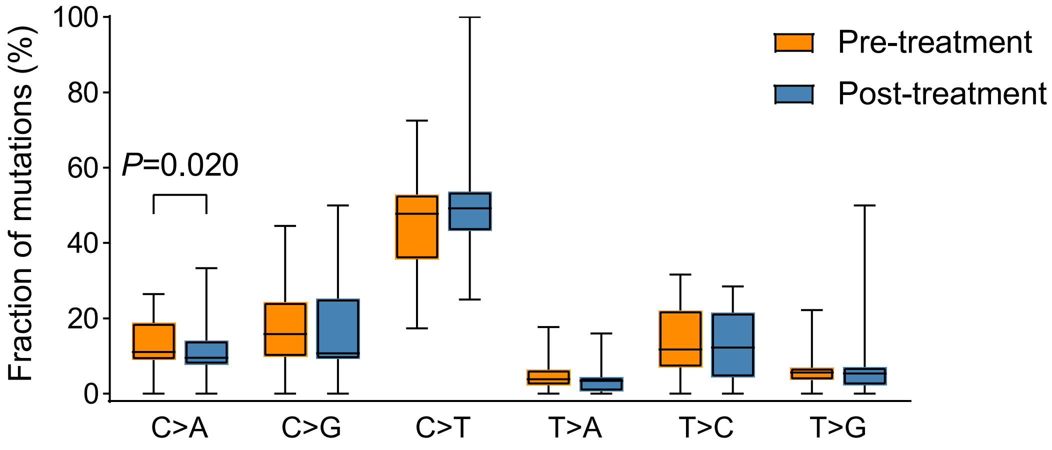

Fig. S2. Comparison of different types of base substitutions in the pre- and post-treatment tumors. *P* values were based on the Wilcoxon signed-rank test.

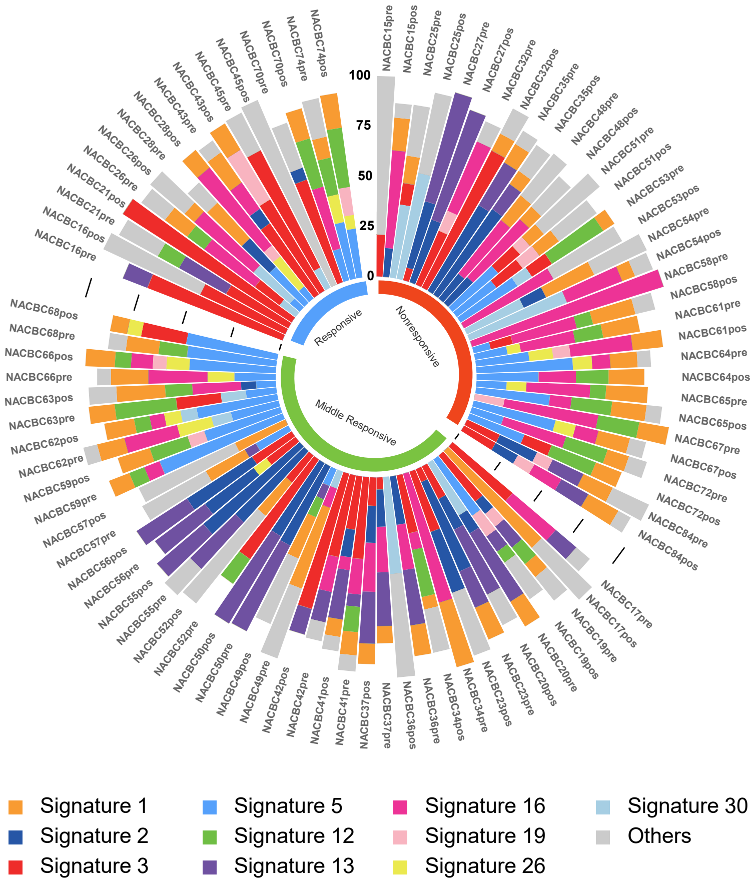

Fig. S3. Distributions of the ten main COSMIC signatures in each pre- and post-treatment tumor.

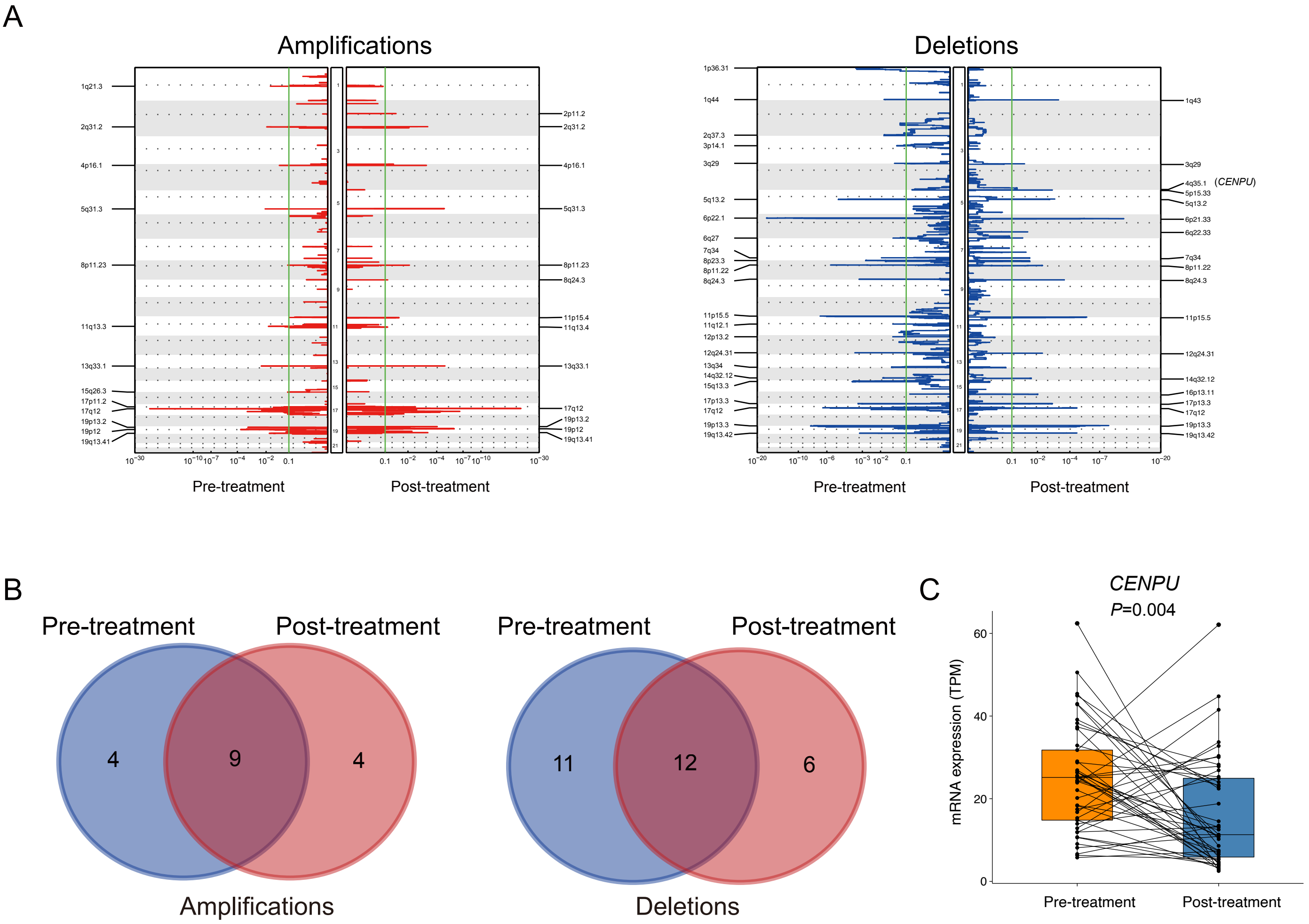

Fig. S4. Copy number alteration between the pre- and post-treatment tumors. (A) SCNA profiles of amplifications (*left panel*) and deletions (*right panel*) identified by the GISTIC2.0 in the pre- and post-treatment tumors. (B) The Venn diagram displays the shared regions in the GISTIC2.0 amplifications and deletions between the pre- and post-treatment tumors. (C) The expression changes of *CENPU* following NAC. *P* values were based on the Wilcoxon signed-rank test.

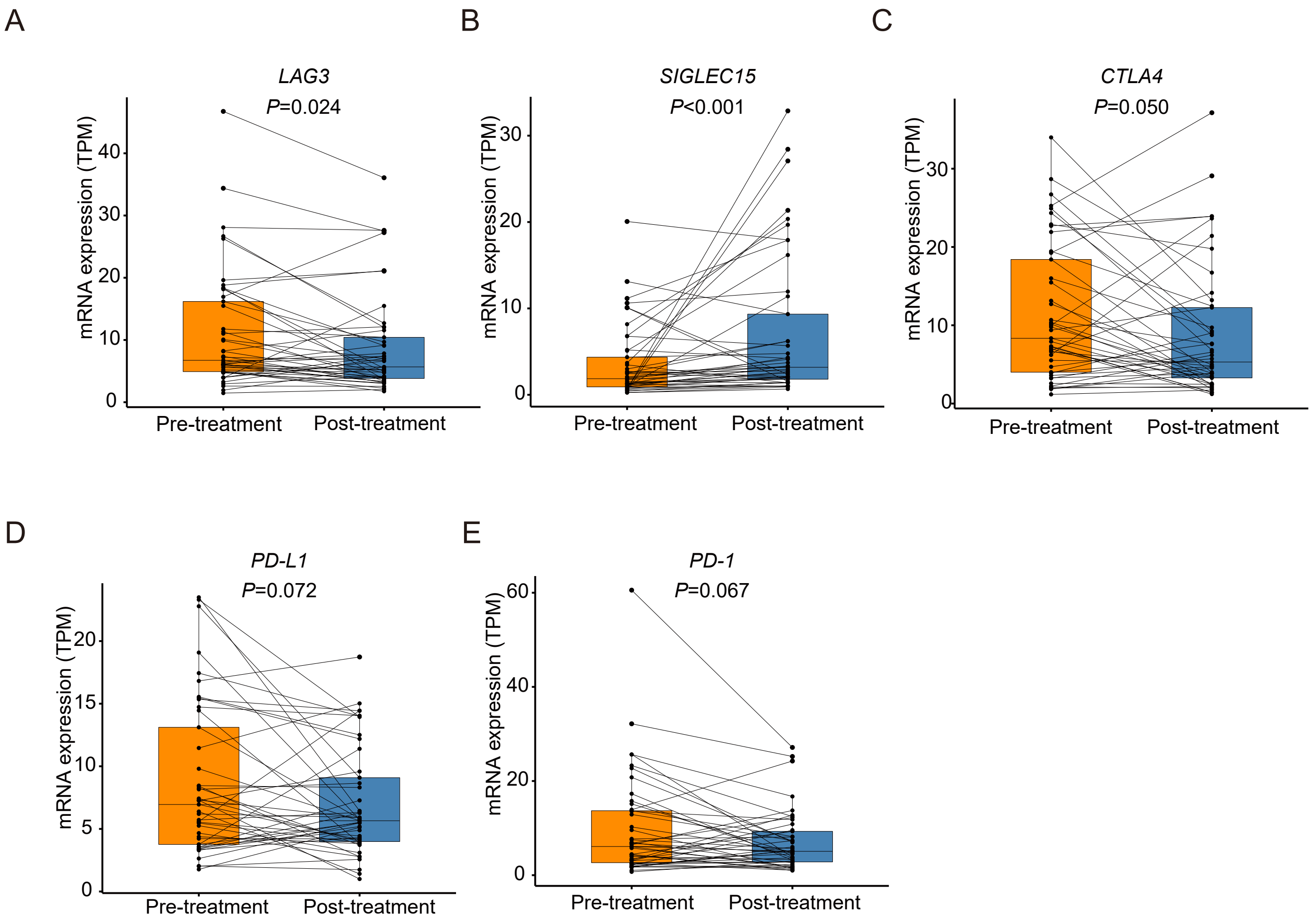

Fig. S5. Changes in immune related gene expression between the pre- and post-treatment tumors. *P* values were based on the Wilcoxon signed-rank test.

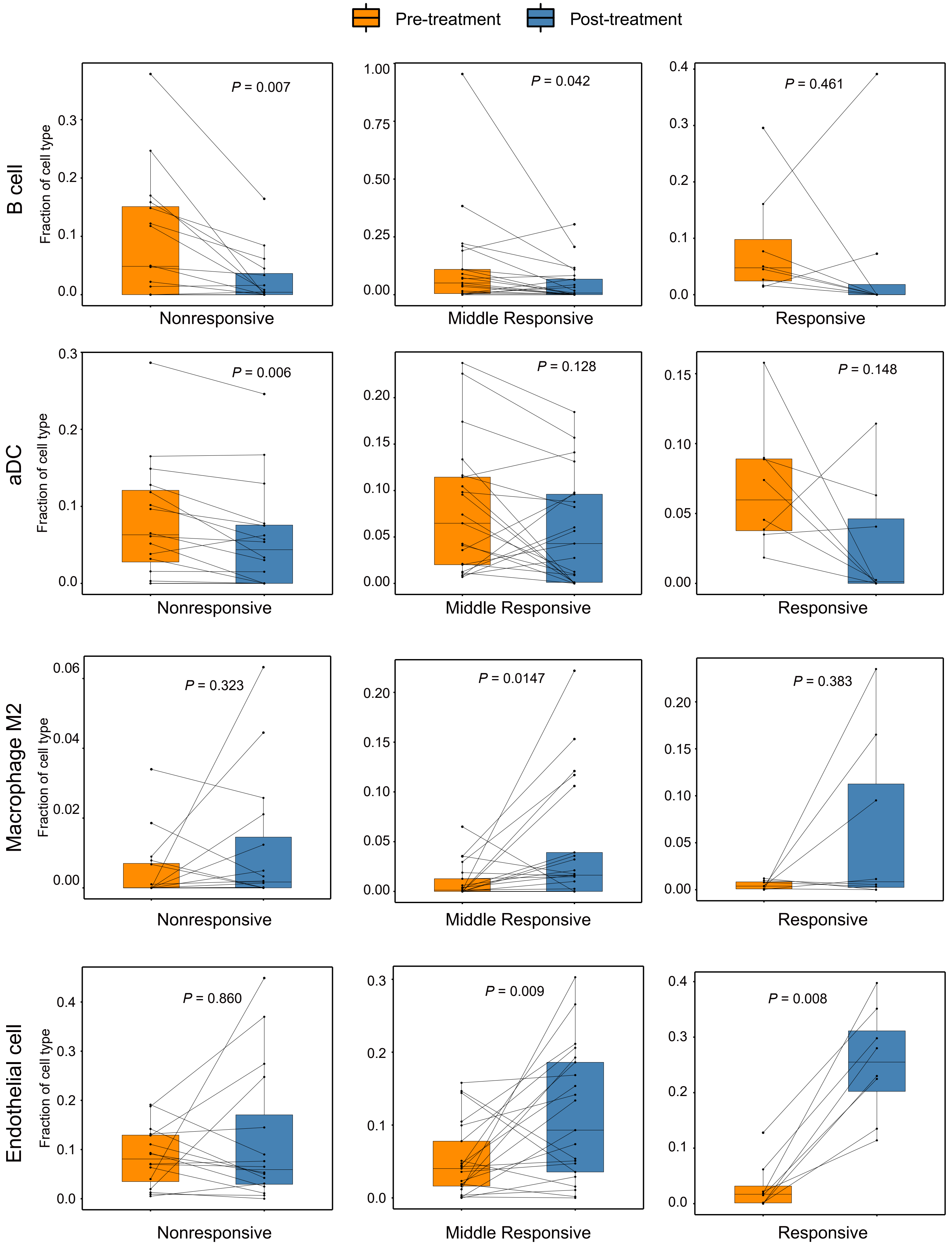

Fig. S6. Changes in the composition of immune and stroma cells between the pre- and post-treatment tumors in different NAC responsive subgroups. *P* values were based on the Wilcoxon signed-rank test.

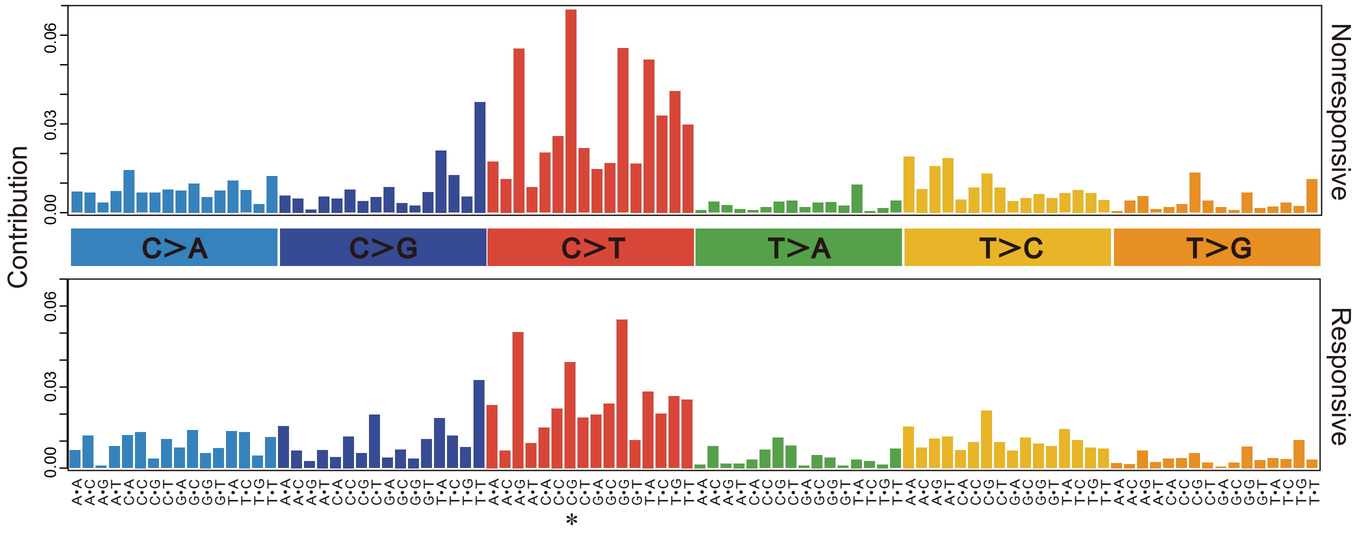

Fig. S7. Comparison of 96 base substitution classification in the pre-treatment tumors of responsive and nonresponsive groups. The Wilcoxon rank sum test was used to compute the *P* values. *$P<0.05$.

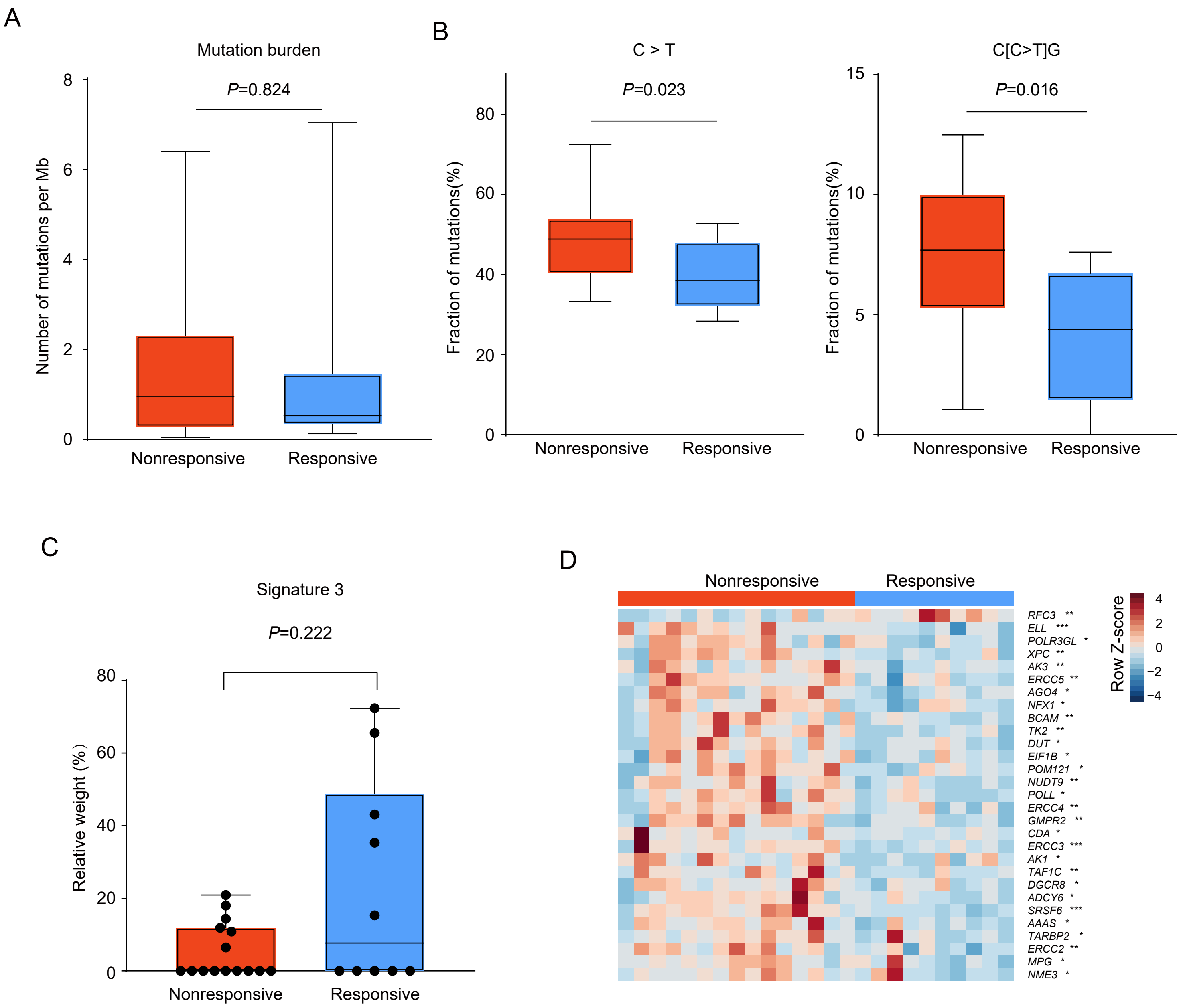

Fig. S8. Mutational signatures in the pre-treatment tumors with samples containing germline mutations removed. Comparison of tumor mutational burden (A) and nucleotide substitutions (B) of C>T (*left*), C[C>T]G (*right*)) between the nonresponsive and responsive groups. (C) Comparison of the relative weights of the COSMIC signature 3 between the nonresponsive and responsive groups. (D) Heatmap comparison of the statistically significant genes related to the DNA repair pathway between responsive and nonresponsive pre-treatment tumors. P values were calculated based on the Wilcoxon rank sum test; ****P*<0.001, ***P*<0.01, **P*<0.05.

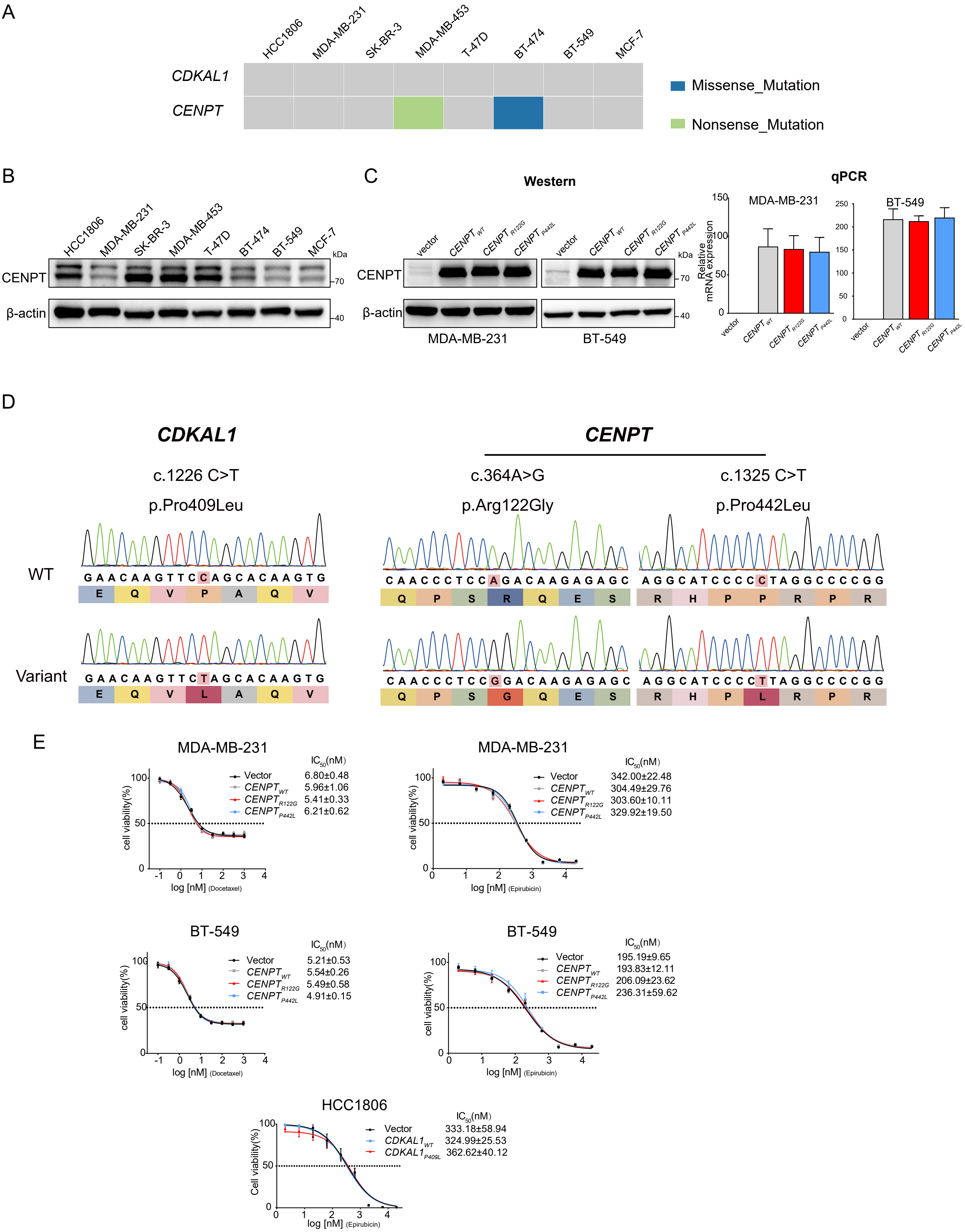
A
HCC1806
MDA-MB-231
SK-BR-3
MDA-MB-453
T-47D
BT-474
BT-549
MCF-7
CDKAL1
CENPT
Missense_Mutation
Nonsense_Mutation
B
CENPT
β-actin
kDa
70
40
C
Western
vector
CENPT WT
CENPT R122G
CENPT P442L
CENPT
β-actin
MDA-MB-231
BT-549
qPCR
Relative mRNA expression
D
CDKAL1
CENPT
c.1226 C>T
p.Pro409Leu
c.364A>G
p.Arg122Gly
c.1325 C>T
p.Pro442Leu
WT
Variant
G A A C A A G T T C C A G C A C A A G T G
E Q V P A Q V
G A A C A A G T T C T A G C A C A A G T G
E Q V L A Q V
C A A C C C T C C A G A C A A G A G A G C
Q P S R Q E S
C A A C C C T C C G G A C A A G A G A G C
Q P S G Q E S
A G G C A T C C C C C T A G G C C C C G G
R H P P R P R
A G G C A T C C C C T T A G G C C C C G G
R H P L R P R
E
MDA-MB-231
IC50(nM)
Vector 6.80±0.48
CENPT WT 5.96±1.06
CENPT R122G 5.41±0.33
CENPT P442L 6.21±0.62
cell viability(%)
log [nM] (Docetaxel)
MDA-MB-231
IC50(nM)
Vector 342.00±22.48
CENPT WT 304.49±29.76
CENPT R122G 303.60±10.11
CENPT P442L 329.92±19.50
log [nM] (Epirubicin)
BT-549
IC50(nM)
Vector 5.21±0.53
CENPT WT 5.54±0.26
CENPT R122G 5.49±0.58
CENPT P442L 4.91±0.15
log [nM] (Docetaxel)
BT-549
IC50(nM)
Vector 195.19±9.65
CENPT WT 193.83±12.11
CENPT R122G 206.09±23.62
CENPT P442L 236.31±59.62
log [nM] (Epirubicin)
HCC1806
IC50(nM)
Vector 333.18±58.94
CDKAL1 WT 324.99±25.53
CDKAL1 P409L 362.62±40.12
Cell viability(%)
log [nM] (Epirubicin)

Fig. S9. Sensitivity of CDKAL1 and CENPT mutation to chemotherapy drugs in BC cells. (A) Distribution of *CDKAL1* and *CENPT* non-synonymous mutations in different breast cancer cell lines based on the CCLE data. (B) Western blot analysis of the expression of CENPT in different human breast cancer cell lines. (C) MDA-MB-231 and BT-549 were infected with $CENPT_{WT}$, $CENPT_{R122G}$ and $CENPT_{P442L}$ overexpressing lentivirus. Western blot and quantitative real-time PCR analyses of CENPT expression levels at the protein and RNA levels. (D) Sanger sequencing chromatograms of PCR-amplified *CDKAL1*, *CENPT* cDNA fragments from $CDKAL1_{WT}$, $\mathrm{CDKAL1}_{P409L}$, $CENPT_{WT}$, $CENPT_{R122G}$ and $CENPT_{P442L}$ cells, and specific nucleotide substitutions (c.1226 C>T, c.364A>G, and c.1325C>T) are depicted in red. (E) Inhibition of proliferation of empty vector, $CENPT_{WT}$, $CENPT_{R122G}$, and $CENPT_{P442L}$ cells (MDA-MB-231 and BT-549) treated with increasing doses of docetaxel and epirubicin. Inhibition of proliferation of empty vector, $CDKAL1_{WT}$ and $CDKAL1_{P409L}$ HCC1806 cells treated with increasing doses of epirubicin. Data are represented as mean ± SD (*n*=3).

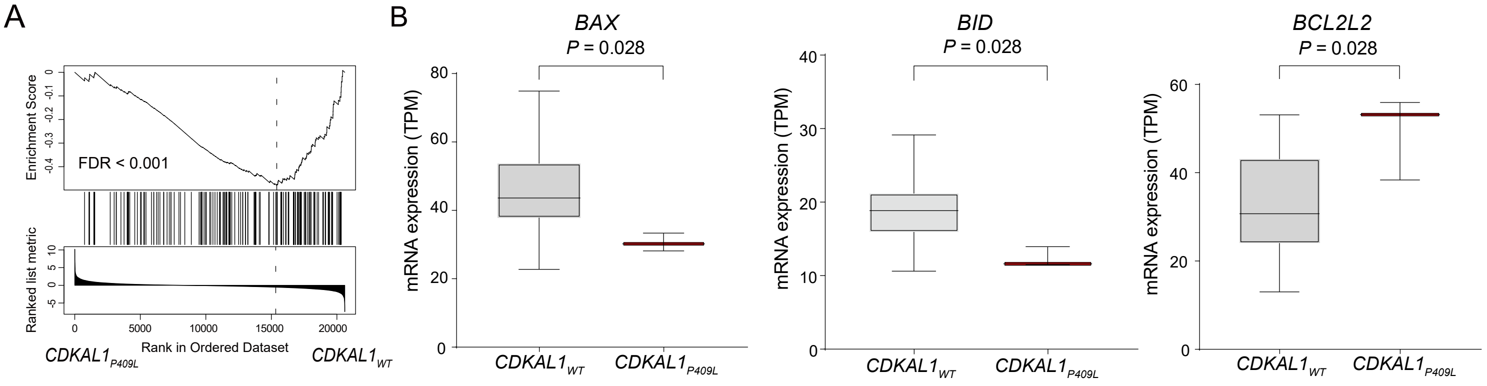

Fig. S10. RNA-seq data analysis between the *CDKAL1*$_{WT}$ and *CDKAL1*$_{P409L}$ tumors. (A) The HALLMARK_APOPTOSIS set was significantly enriched in the *CDKAL1*$_{WT}$ tumors relative to *CDKAL1*$_{P409L}$ mutant tumors. FDR was based on the gene set enrichment analysis. (B) *BAX*, *BID* and *BCL2L2* mRNA expression levels in the *CDKAL1*$_{WT}$ and *CDKAL1*$_{P409L}$ groups. The Wilcoxon rank sum test was used to compute the *P* values.

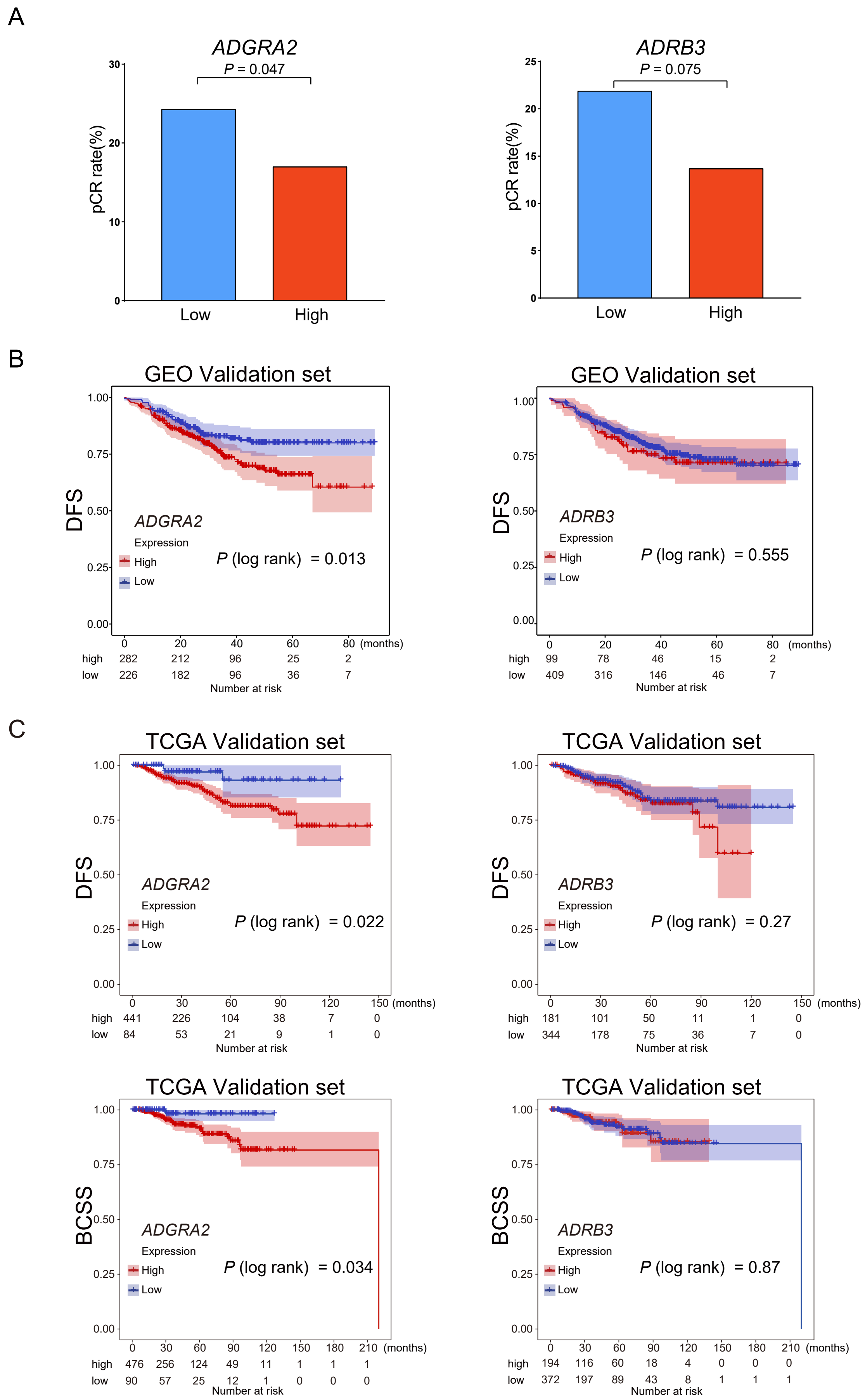

Fig. S11. Associations between *ADGRA2* or *ADRB3* expression and pCR or prognosis of BC patients. (A) Bar graph comparing the pCR rates between groups of BC patients with low and high expression levels of *ADGRA2* and *ADRB3* in the GEO validation set. Pearson's chi-square test was used to compute the *P* values. Kaplan-Meier DFS or/and BCSS analyses in the GEO validation set (B) and the TCGA validation set (C) based on high and low expression of *ADGRA2* and *ADRB3*. *P* values were calculated based on the log-rank test.
